# Indicator system for regional public health emergency response capacity based on ternary space theory: construction and empirical study in China

**DOI:** 10.1186/s12889-026-28703-4

**Published:** 2026-08-01

**Authors:** Jian Wu, Zi-an Zhou, Dong-fu Qian, Meng-bing Du, Jing Yang

**Affiliations:** 1 Laboratory for Digital Intelligence & Health Governance, Nanjing Medical University, Nanjing, Jiangsu 211166 China; 2 School of Health Policy and Management, Nanjing Medical University, Nanjing, Jiangsu 211166 China; 3 Institute of Strategic Studies, University of Chinese Academy of Sciences, Beijing, 100190 China; 4 School of Political Science and Public Administration, Wuhan University, Wuhan, Hubei 430072 China

**Keywords:** Regional public health emergency response capacity, Ternary space theory, Game theory combination weighting, SHAP value method

## Abstract

**Background:**

Establishing a comprehensive national public health emergency management system is essential for ensuring population health. However, pronounced disparities in economic development and healthcare resources among China’s provinces have led to heterogeneous public health emergency response capabilities, highlighting the need for an evaluation framework that incorporates region-specific features.

**Methods:**

Based on the ternary space theory, an indicator system was constructed across physical, social, and information dimensions. Provincial panel data from 2013 to 2021 were analyzed using game theory-based combination weighting and TOPSIS methods. SHAP analysis was employed to identify key influencing factors.

**Results:**

Emergency response capacity improved overall but exhibited significant spatial variation, with eastern regions outperforming western areas, and a structural imbalance whereby pre-preparation in most provinces lagged behind response and recovery capacities. Regional economic development and transportation infrastructure density were identified as the most critical determinants.

**Conclusions:**

The findings emphasize the necessity of optimizing resource allocation, enhancing medical agglomeration in underserved regions, and strengthening transportation networks to mitigate regional disparities and improve national public health resilience.

**Supplementary Information:**

The online version contains supplementary material available at https://doi.org/10.1186/s12889-026-28703-4.

## Introduction

The accelerating pace of globalization has elevated public health emergencies to a critical determinant of national security, social stability, and population health outcomes [1]. Recent decades have witnessed a series of escalating global health crises, including the Severe Acute Respiratory Syndrome (SARS) outbreak in 2003, the influenza A (H1N1) pandemic in 2009, the H7N9 avian influenza epidemic in 2013, and the unprecedented COVID-19 pandemic in 2020, which have collectively exposed systemic vulnerabilities in global public health infrastructure [2]. In response to these challenges, the “Proposal of the Central Committee of the Communist Party of China on Formulating the Fourteenth Five-Year Plan for National Economic and Social Development and the Long-Range Objectives Through the Year 2035” has explicitly prioritized the enhancement of public health emergency response capabilities as a strategic imperative. This policy directive underscores the paramount importance accorded by the Party and state to strengthening national public health preparedness. Consequently, the development of effective strategies for improving public health emergency response capacity has emerged as a critical component in advancing national governance capabilities and ensuring sustainable development.

As a vast nation with a large population, complex topography, and diverse regional cultures, China’s uneven regional development not only influences economic and social domains but also extends, to some extent, into the realm of public health. Significant disparities exist among different regions in China in terms of economic development levels, medical and health conditions, and natural geographic environments [3]. These disparities pose considerable challenges to establishing unified coordination mechanisms and strategic planning for nationwide public health emergency responses. Regions with weaker health infrastructure and limited information dissemination capacity face higher risks of morbidity and mortality during outbreaks or disasters. Therefore, assessing the public health emergency response capabilities of different regions and identifying the underlying factors that constrain their development is crucial for improving the capacity to respond to public health emergencies. However, existing assessment frameworks predominantly focus on the micro-level of organizations or individuals. When conducting regional-level evaluations, these systems often fail to systematically incorporate key regional characteristics such as geographical area into the indicator framework. This gap limits the accuracy of cross-regional capacity comparisons and hinders systemic improvements. To address this limitation, the present study develops a comprehensive evaluation framework grounded in ternary space theory, aiming to provide both theoretical and practical insights for strengthening China’s public health emergency management system.

The principal innovations and contributions of this paper are threefold: (a) We introduce a novel methodological framework for indicator weight determination through a comprehensive combinatorial weighting approach, thereby advancing extant methodologies in weight assignment techniques. (b) This paper develops a comprehensive evaluation index system for regional public health emergency response capacity, grounded in ternary space theory. Regional heterogeneity is quantified through agglomeration indices and per-capita normalization, embedded as structural indicators within ternary dimensions during weight generation. This innovative framework not only expands the existing repertoire of evaluation metrics and methodologies for assessing public health emergency preparedness but also provides a novel theoretical perspective for analyzing emergency response capabilities. (c) Employing longitudinal provincial panel data spanning 2013 to 2021, we conduct robust empirical analyses that elucidate critical deficiencies and regional disparities in public health emergency response capacities. (d) Through the application of Shapley Additive Explanations (SHAP) value analysis, we identify and quantify the key constraining factors impeding the development of regional public health emergency response capabilities, offering valuable insights for targeted policy interventions.

## Literature review

### Evaluation of emergency management capacity for public health emergencies

Research on public health emergencies began earlier in foreign countries, while in China, extensive studies on emergency response capacity for public health emergencies were initiated following the 2003 SARS outbreak [4]. These studies have primarily focused on three areas: the construction of emergency management systems, the development of emergency management capacity, and the evaluation of emergency management capacity [5]. Among these, the evaluation of emergency response capacity for public health emergencies has been discussed mainly from three levels: regional-level assessments, institutional capacity evaluations, and individual competency analyses.

1. Regional-Level Systems: including national, provincial, and other regions. For instance, The United States has developed the National Health Security Preparedness Index (NHSPI) [6] to comprehensively assess its preparedness for public health emergencies across six key domains: health security surveillance, community planning and engagement, incident and information management, healthcare delivery, countermeasure management, and environmental and occupational health. Li and Gu [7] conducted a study based on 31 provinces in China, constructing an evaluation index system grounded in the emergency management process theory to assess emergency response capacity for public health emergencies.
2. Institutional-Level Systems: mainly the government, healthcare institutions, and grassroots communities. Al-Hajj et al. [8] utilized the Delphi method and developed a Hospital Disaster and Emergency Preparedness Tool to evaluate the level of disaster and emergency preparedness in hospitals. An et al. [9] constructed an evaluation system for emergency management capacity in rural public health emergencies, which includes four dimensions: emergency entities, emergency mechanisms, resources, and concepts.
3. Individual-Level Systems: covering healthcare personnel, community personnel, etc. Wang et al. [10] conducted an assessment of emergency preparedness capabilities among public health personnel from 84 rural disease control and prevention centers in China through anonymous questionnaires and semi-structured interviews. Additionally, Zhou et al. [11] assessed the awareness, attitudes, behaviors, and response capabilities of primary healthcare workers in Guangdong Province, China, towards public health emergencies through a questionnaire survey.

In the establishment of evaluation index systems, there are numerous approaches to categorize indicators. For instance, Hao et al. [12] integrated resilience theory with the theory of balanced emergency management throughout the process, selecting 31 evaluation indicators across six key dimensions: preparedness, early warning, mitigation, response, recovery, and learning. A TOPSIS model was constructed to assess the emergency management capacity for public health emergencies in 18 cities of Henan Province. Xu et al. [13] established an index system encompassing four dimensions- collaborative cooperation, medical and health security, socio-economic stability, and infrastructure construction-and employed the entropy method to conduct a comprehensive quantitative assessment of public health emergency management capacity. Lu et al. [5] developed an evaluation index system for grassroots community public health emergency management capacity organized around four dimensions: organizational identity, competency identity, advanced capability identity, and emotional identity.

Through a comprehensive analysis of these indicators across multiple dimensions, it becomes apparent that all metric categories can be systematically classified into three distinct phases corresponding to the temporal progression of incident development: preparedness, response, and recovery phases. At the regional level of analysis, the extant literature reveals a notable gap in the incorporation of region-specific characteristics and contextual factors into the development of such evaluation frameworks.

### Analysis of factors influencing public health emergency response capacity

Scholars both domestically and internationally have conducted extensive research on the factors influencing public health emergency response capacity, exploring and analyzing these factors from two perspectives: external environmental factors and internal influencing factors. The study by Ning et al. [14] indicates that vague responsibility allocation, lack of clear national job requirements, and insufficient knowledge and skill levels among personnel are the main issues hindering the improvement of preparedness capabilities of public health inspectors. Additionally, Uhm et al. [15] conducted a descriptive cross-sectional study on the disaster response capabilities of 1,020 emergency medical technicians in four South Korean cities. The study found that perception of disaster risk, disaster self-efficacy, experience in disaster education, and personal disaster preparedness are key factors influencing their disaster response capabilities. Some scholars have empirically tested these factors using a range of analytical methods. For instance, Zhao et al. [16] used descriptive analysis and multivariate logistic regression to identify that proficiency in emergency preparedness planning, more practical experience in emergency response, effectiveness of training and drills, higher education levels, and higher professional positions are significantly associated with the field response capabilities of Center for Disease Control and Prevention (CDC) professionals. However, public health emergency management capacity is inherently complex, and its formation pathways are diverse. As a result, its construction and manifestation are influenced by a multitude of intertwined factors. Therefore, it is necessary to assess the characteristic importance of different influencing factors.

In summary, while existing literature provides valuable theoretical foundations, several critical limitations warrant further investigation:

- (a) current evaluation frameworks predominantly concentrate on micro-level organizational or individual analyses, with limited attention to macro-level regional development perspectives. Furthermore, existing assessment systems frequently neglect regional characteristics, such as regional area, economic development level, and population size, and the inherent heterogeneity and complexity across different geographical areas, thereby compromising the scientific rigor and accuracy of cross-regional emergency response capacity evaluations.
- (b) the investigation of influencing factors predominantly employs qualitative methods or conventional regression models, which exhibit limited explanatory power and fail to adequately capture nonlinear relationships among various determinants.
- (c) while public health emergencies inherently operate within a “ternary space” framework, encompassing physical, social, and information dimensions, current research has insufficiently explored the application of ternary space theory. Notably, no scholarly work has yet systematically applied this theoretical framework to public health emergency response capacity evaluation.

To address these research gaps, this paper introduces a novel framework for constructing a regional public health emergency response capacity evaluation system based on ternary space theory. Additionally, we employ the SHAP value method to analyze the determinants of regional public health emergency response capacity development, complemented by empirical analysis using comprehensive datasets.

## Materials and methods

To provide a comprehensive understanding of the research process, we have included a detailed description of the methods used throughout the study. The methodology encompasses construction of a comprehensive indicator system based on ternary space theory, data collection from authoritative sources, determination of indicator weights using a combination of subjective and objective methods (AHP, CRITIC, and entropy weight methods), and application of the TOPSIS method for regional analysis. Additionally, the SHAP value method was employed to analyze the importance of various factors influencing emergency response capacities.

### Construction of the indicator system

Human living spaces have grown from the original dual composition of physical and social spaces to a triad of interdependent and interacting physical, social, and information spaces due to the in-depth development of information technology [17, 18]. Public health emergencies are sudden, pervasive health threats involving large-scale health prevention and control systems and emergency medical service systems, and are intimately related to social, economic, and information networks [19]. Consider the COVID-19 pandemic as an example. When a sudden, large-scale infectious disease breaks out globally, the ability of healthcare systems to provide the rapidly rising demand for healthcare is severely hampered, and the emergency capacity of public health emergencies is challenged. At the same time, all types of negative news travelled swiftly through the information space, exacerbating public terror and anxiety, and most areas of human social activity had to come to a temporary halt, disrupting the regular order of social life [20] .

**Fig. 1:**
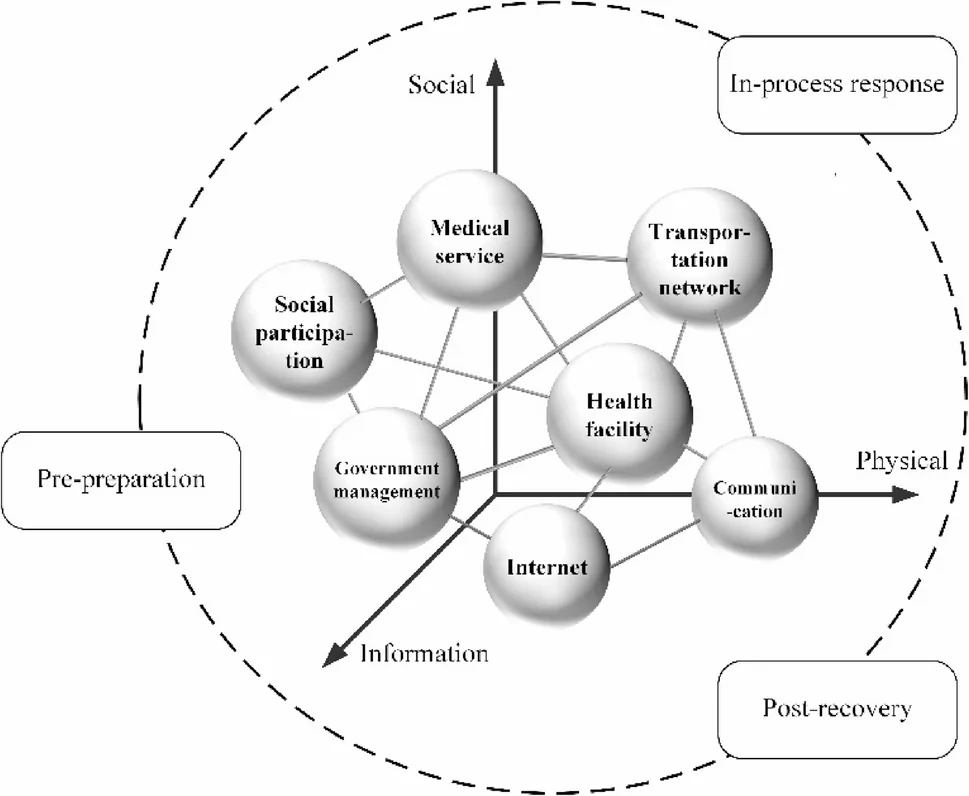
Ternary spatial model of public health emergencies

As illustrated in Fig. 1, the ternary space model for public health emergencies captures the complex interactions among these dimensions. Physical space serves as the material foundation of emergency response capacity, encompassing the allocation of tangible resources such as healthcare institutions, while also providing the operational carrier for the social and information spaces. Social space, acting as a bridge connecting the physical and information spaces, establishes norms for action through social management and services [21]. Information space, focusing on information dissemination and decision-making support, thereby facilitates the coordination of social forces and promotes optimal allocation of physical-space emergency resources.

Based on the theoretical framework above and supported by relevant literature [17, 18, 21], the evaluation indicators were systematically deconstructed into the three spatial dimensions. Indicators related to tangible infrastructure and resource allocation, such as the number of hospital beds and transportation density, were categorized under the physical space. Those reflecting social governance, human resources, and public engagement, such as health education activities and personnel allocation, were assigned to the social space. Indicators associated with information dissemination and technological support, including internet penetration rate and the proportion of science and technology expenditure, were classified into the information space. This classification scheme was further reviewed and validated by a panel of five experts in public health and emergency management to ensure its conceptual rationality and practical applicability.

In addition, to incorporate regional characteristics into the indicator system, this study introduces the concept of public health resource agglomeration degree [22], integrating regional area into the indicators to account for geographical disparities in the distribution of medical and health institutions. Furthermore, population size and economic development level were incorporated into other indicators, thereby reflecting regional differences in responding to public health emergencies.

Using the keywords “public health emergencies” and “indicator system”, we searched database include China National Knowledge Infrastructure and Web of Science, screening 161 high-quality articles that were closely linked to the topic of this paper. Based on existing research findings and references to legal documents such as the Emergency Response Law of the People’s Republic of China and the Emergency Regulations Regarding Public Health Emergencies, and guided by the ternary spatial theory and the three-stage emergency management theory, eventually, we established a regional emergency response capacity evaluation indicator system for public health emergencies, which consists of 44 three-level indicators, as shown in Table 1.

**Table 1:** Indicator system for evaluation of regional public health emergency response capacity

Level 1 indicators	Level 2 indicators	Level 3 indicators	Explanation of Index Calculation
Pre-preparation	Physical space	Centers for Disease Control and Prevention(CDC) per 10,000 population(+)	The number of Centers for Disease Control and Prevention (CDC) per 10,000 permanent residents at the end of the year
		Agglomeration degree of CDC(+)	The ratio of the number of CDCs in the region to the total land area of the region / The ratio of the number of CDCs in the country to the total land area of the country
		Share of health supervision institute in professional public health institutions(+)	(The number of health supervision institutes / The number of professional public health institutions) × 100%
		Share of level A tertiary hospitals in the number of hospitals(+)	(The number of Grade A tertiary hospitals / The total number of hospitals) × 100%
		Number of beds in preventive health departments per 10,000 population(+)	The number of beds in preventive health departments of hospitals per 10,000 permanent residents at the end of the year
		Agglomeration degree of beds in preventive health departments(+)	The ratio of the number of beds in preventive health departments in the region to the total land area of the region / The ratio of the number of beds in preventive health departments nationwide to the total land area of the country
	Social space	CDC personnel per 10,000 population(+)	The number of personnel in Centers for Disease Control and Prevention (CDC) per 10,000 permanent residents at the end of the year
		Agglomeration degree of CDC personnel(+)	The ratio of the number of CDC personnel in the region to the total land area of the region / The ratio of the number of CDC personnel nationwide to the total land area of the country
		Annual health check-ups as a proportion of the population(+)	(The number of people undergoing health check-ups at medical and health institutions / The number of permanent residents at the end of the year) × 100%
		Number of promotional materials per 10,000 population(+)	The promotional materials include print materials, audio-visual products, mobile text messages, and physical items.
		Number of public health education activities per 10,000 population(+)	/
		Share of medical expenditure in fiscal expenditure(+)	The fiscal expenditure data are derived from the general budget expenditure recorded in the statistical yearbook.
		Number of family health services per 10,000 population(+)	/
	Information space	Coverage of websites hosted by health education professional bodies(+)	(The number of websites hosted by health education professional bodies / The number of permanent residents at the end of the year) × 100%
		Health Education Column Coverage(+)	(Health education column count / Permanent resident population at year - end) × 100%
In-process response	Physical space	Number of emergency medicine beds per 10,000 population(+)	/
		Number of infectious disease beds per 10,000 population(+)	/
		Hospitals per 10,000 population(+)	/
		Agglomeration degree of hospital(+)	/
		Number of beds in other hospital departments per 10,000 population(+)	/
		Number of beds in primary healthcare institutions per 10,000 population(+)	/
		Number of primary healthcare institutions as a proportion of the number of grassroots organizations in urban and rural areas(+)	/
		Transport network density(+)	/
	Social space	The utilization rate of hospital beds(-)	/
		The daily average workload of patient visits of physicians in the hospital(-)	/
		Primary healthcare institutions personnel per 10,000 population(+)	/
		Agglomeration degree of personnel in primary healthcare institutions(+)	/
		Hospital practicing physician (assistants) per 10,000 population(+)	/
		Hospital registered nurses per 10,000 population(+)	/
		Agglomeration degree of personnel in hospital(+)	/
		Strength of leadership of grassroots organizations(+)	The ratio of the total population to the number of social self-governance organizations (including sub-districts, townships, and village committees)^[13]^
	Information space	Internet penetration rate(+)	/
		Telephone penetration rate(+)	/
Post-recovery	Physical space	Rehabilitation medicine beds per 10,000 population(+)	/
		Agglomeration degree of beds in Rehabilitation medicine departments(+)	/
		Psychiatric beds per 10,000 population(+)	/
		Agglomeration degree of beds in Psychiatric departments(+)	/
	Social space	Share of urban residents’ healthcare expenditure in consumer expenditure(+)	/
		Share of rural residents’ healthcare expenditure in consumer expenditure(+)	/
		Number of technical consultations and policy recommendations(+)	/
		Number of medical aid funds per capita for subsidized persons(+)	Total amount of medical aid funds / Total number of subsidized persons
		The incidence rate of class A and B notifiable infectious diseases (1/100,000) (-)	/
		The mortality rate of class A and B notifiable infectious diseases (1/100,000) (-)	/
	Information space	Share of science and technology expenditures in fiscal expenditures(+)	/

Positive indicators (+) indicate that a higher value of the indicator corresponds to stronger emergency response capacity, while negative indicators (-) indicate that a higher value of the indicator corresponds to weaker emergency response capacity

The evaluation system covers the entire emergency management process, comprising: (a) Preparation phase indicators, such as disease prevention [23] and health education and public awareness campaigns [24], and medical expenditure allocation [13] ; (b) Response phase indicators, including emergency mobilization speed, resource distribution efficiency [25], and information dissemination mechanisms [26] ; (c) Recovery phase indicators, such as medical rehabilitation programs [27], economic revitalization initiatives, and institutional learning from crisis experiences [7]. Among them, the utilization rate of hospital beds, the daily average workload of patient visits of physicians in the hospital, the incidence rate of class A and B notifiable infectious diseases (1/100,000), and the mortality rate of class A and B notifiable infectious diseases (1/100,000) are negative indicators.

### Data sources

The study focuses on the emergency response capacity for public health emergencies in 31 provinces in China (excluding Hong Kong, Macao, and Taiwan), with provincial panel data used to investigate regional emergency response capacity. The data were acquired from authoritative statistics issued by the National Bureau of Statistics, *China Statistical Yearbook* (2014–2022), and *China’s Hygiene and Health Statistical Yearbook* (2014–2022), all of which are highly credible and comparable.

In the data preprocessing phase, we first employed the boxplot method to identify outliers in the data. Subsequently, we replaced these outliers and filled in the time-discontinuous and missing data using linear interpolation, thereby ensuring the integrity and accuracy of the data.

### Methods for determining indicator weights and evaluation models

Evaluation indicator weights reflect their relative importance in assessment outcomes [28], playing a crucial role in evaluation research. Weight determination methods generally fall into two categories: subjective and objective approaches [29] .Subjective methods [30, 31], such as the Analytic Hierarchy Process (AHP) and expert scoring, rely on decision-makers’ expertise. Objective methods [32], including entropy weight and CRITIC (Criteria Importance Through Intercriteria Correlation) approaches, derive weights directly from data, minimizing subjective bias. Given the complex interrelationships and inherent subjectivity in public health emergency evaluations, researchers have increasingly adopted combined weighting methods. The game theory-based approach has proven particularly effective, as it reconciles conflicting weighting methods through optimization, achieving balanced decision-making outcomes [33] .

Building on the discussion of weight determination methods, it is also important to consider the different types of evaluation models used in public health emergency response capacity assessments. Evaluation models can be categorized into qualitative and quantitative approaches. Qualitative evaluation methods include the Delphi method, factor analysis and others [34, 35], while quantitative evaluation methods encompass the TOPSIS method, Data Envelopment Analysis (DEA) [36], and evaluation models based on artificial intelligence [37]. For instance, Qian et al. [38] constructed a post-popularity evaluation model based on the TOPSIS entropy weight method and used K-means clustering to build user profiles in online health communities during public health emergencies, demonstrating the application of quantitative methods in multi-criteria decision-making. In the assessment of public health emergencies, quantitative methods are widely adopted due to their precision and operability.

Therefore, in this study, we employ the game theory-based combination weighting method to integrate subjective weights derived from AHP with objective weights obtained through CRITIC and entropy methods. This approach effectively balances expert judgment with data-driven insights, minimizing the limitations of single weighting techniques. Following weight determination, the TOPSIS method is applied to evaluate and rank the public health emergency response capacity of each province. TOPSIS is chosen for its ability to handle multiple criteria and provide clear comparative results based on proximity to ideal solutions [39]. The computational details of the game theory-based combination weighting approach, along with the AHP, CRITIC, entropy, and TOPSIS methods, are provided in Appendix A.

### SHAP feature importance assessment model

The SHAP model is an additive feature attribution approach that calculates the marginal contribution of features to the model output. In this method, all features are regarded as “contributors” [40]. By utilizing the SHAP model, it is possible to quantify the influence of different features on the results, identify the most important influences, and have a high degree of interpretability [41]. The SHAP model [42] is calculated as below

1 $${\phi _k}=\sum\nolimits_{{S \subseteq R\backslash k}} {\frac{{\left| S \right|!\left( {\left| R \right| - \left| S \right| - 1} \right)!}}{{\left| R \right|!}}\left[ {F\left( {S \cup \left\{ k \right\}} \right) - F\left( S \right)} \right]} $$

Based on the additive feature approach, the linear function $g\left( {{x_i},{\eta _i}} \right)$ is defined as

2 $$g\left( {{x_i},{\eta _i}} \right)=H\left( {\delta _{i}^{k}} \right)={\phi _0}+\sum\nolimits_{{k=1}}^{K} {{\phi _k}\delta _{i}^{k}} $$

## Results

### Calculation of indicator weights

Based on the method proposed in the previous subsection, the overall process of calculating the weights of indicators in this paper is as follows: when seeking subjective weights, the indicators at all levels were drawn into a questionnaire, and five experts in the field of public health and emergency management were invited to score the importance of the indicators based on Table 1. The scoring data were collated to calculate the subjective weights using the AHP method.

When seeking objective weights, we used the 2013–2021 provincial data to construct a judgment matrix, applied the step-transformation method to unify direction and remove scale effects, non-negatively shifted the results to obtain a standardized matrix, calculated CRITIC and entropy weights separately, and finally combined them via game-theory weighting. The results of the assignment of indicators at all levels are shown in Table B1 in Appendix B. The weight information in parentheses of the first-level and second-level indicators in the table is the subjective assignment result based on the AHP method.

### Horizontal TOPSIS sorting

#### Analysis of the current status of regional public health emergency response capacity

Through the weight information of the game theory combination weighting method and the TOPSIS-based ranking method in "Horizontal TOPSIS sorting" section, the relative proximity of the emergency response capacity of public health emergencies in each province from 2013 to 2021 was calculated, and the specific results are shown in Table 2.

**Table 2:** Evaluation results of public health emergency response capacity by province

Province	2013	2014	2015	2016	2017	2018	2019	2020	2021	Average value
Beijing	0.525	0.531	0.517	0.510	0.526	0.518	0.511	0.494	0.446	0.509
Tianjin	0.386	0.386	0.386	0.387	0.378	0.375	0.362	0.348	0.319	0.370
Hebei	0.234	0.249	0.239	0.242	0.255	0.265	0.263	0.278	0.252	0.253
Shanxi	0.279	0.279	0.278	0.267	0.263	0.259	0.261	0.267	0.261	0.268
Inner Mongolia	0.284	0.288	0.295	0.297	0.294	0.293	0.299	0.284	0.298	0.292
Liaoning	0.309	0.312	0.320	0.308	0.315	0.308	0.298	0.271	0.262	0.300
Jilin	0.226	0.264	0.236	0.243	0.252	0.260	0.264	0.261	0.256	0.251
Heilongjiang	0.229	0.242	0.252	0.252	0.267	0.275	0.303	0.292	0.301	0.268
Shanghai	0.636	0.622	0.610	0.612	0.623	0.620	0.608	0.606	0.567	0.612
Jiangsu	0.326	0.325	0.322	0.322	0.327	0.334	0.334	0.316	0.332	0.326
Zhejiang	0.346	0.347	0.353	0.354	0.342	0.340	0.342	0.313	0.342	0.342
Anhui	0.217	0.225	0.232	0.271	0.273	0.291	0.312	0.320	0.320	0.273
Fujian	0.301	0.311	0.310	0.294	0.292	0.287	0.281	0.264	0.240	0.287
Jiangxi	0.196	0.217	0.222	0.213	0.232	0.252	0.282	0.299	0.301	0.246
Shandong	0.291	0.312	0.303	0.296	0.309	0.319	0.328	0.308	0.306	0.308
Henan	0.225	0.240	0.238	0.242	0.251	0.259	0.272	0.280	0.354	0.262
Hubei	0.228	0.250	0.258	0.265	0.286	0.304	0.320	0.325	0.283	0.280
Hunan	0.202	0.212	0.211	0.218	0.223	0.240	0.252	0.263	0.239	0.229
Guangdong	0.338	0.334	0.353	0.363	0.364	0.380	0.378	0.347	0.308	0.352
Guangxi	0.176	0.181	0.182	0.178	0.180	0.186	0.183	0.200	0.180	0.183
Hainan	0.228	0.230	0.235	0.254	0.228	0.227	0.237	0.237	0.279	0.239
Chongqing	0.213	0.222	0.241	0.245	0.255	0.264	0.266	0.262	0.240	0.245
Sichuan	0.177	0.191	0.203	0.211	0.223	0.231	0.250	0.249	0.259	0.222
Guizhou	0.182	0.208	0.219	0.218	0.241	0.250	0.259	0.256	0.267	0.233
Yunnan	0.167	0.190	0.193	0.197	0.209	0.223	0.213	0.215	0.225	0.204
Tibet	0.331	0.321	0.335	0.329	0.347	0.337	0.337	0.327	0.393	0.340
Shaanxi	0.235	0.261	0.274	0.257	0.266	0.267	0.273	0.272	0.259	0.263
Gansu	0.204	0.204	0.221	0.209	0.221	0.231	0.232	0.207	0.229	0.218
Qinghai	0.323	0.335	0.366	0.362	0.351	0.343	0.361	0.360	0.379	0.353
Ningxia	0.240	0.234	0.255	0.270	0.284	0.276	0.263	0.249	0.260	0.259
Xinjiang	0.257	0.252	0.270	0.265	0.259	0.279	0.277	0.282	0.326	0.274

**Fig. 2:**
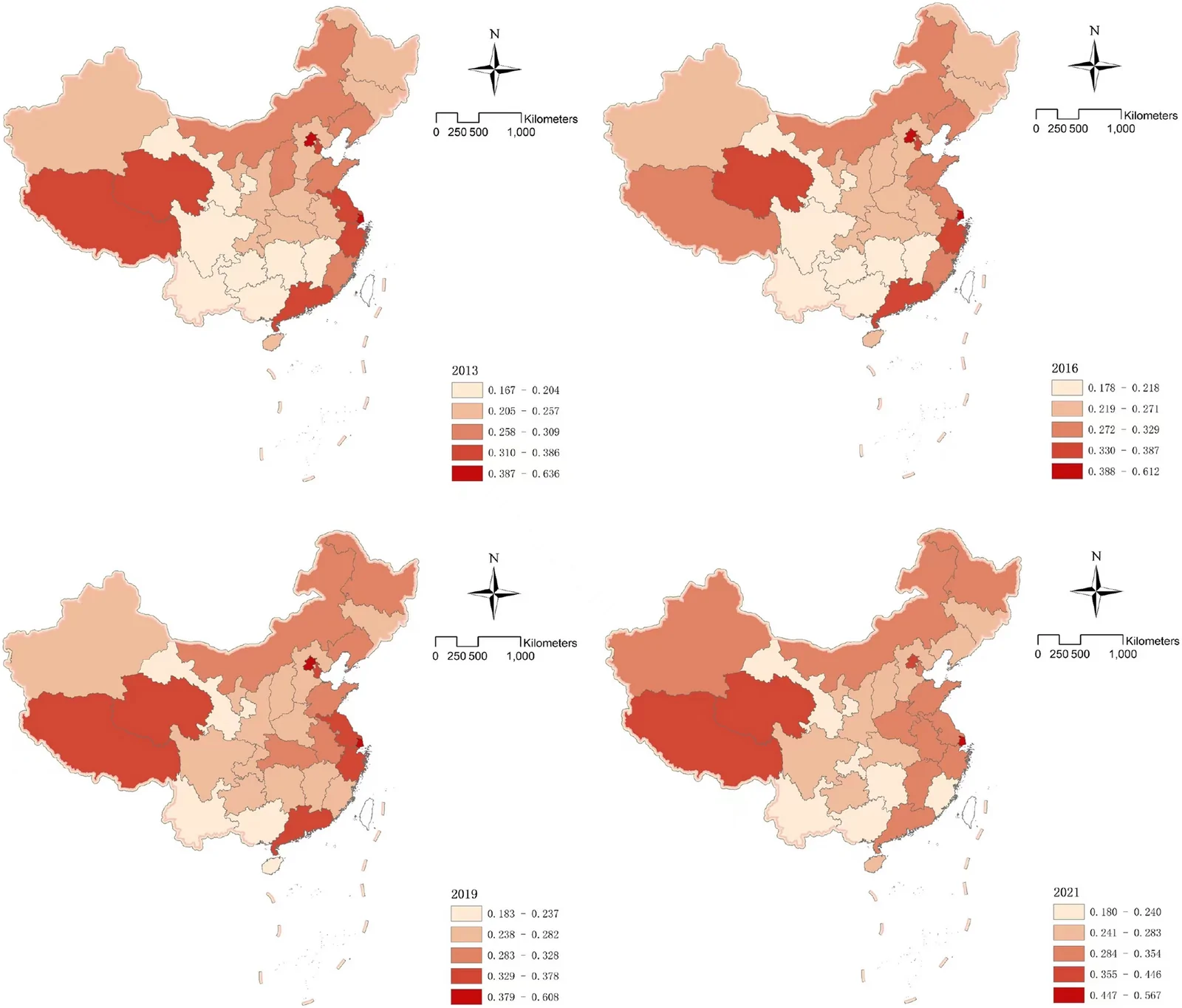
Spatial distribution of regional public health emergency response capacity by province

**Fig. 3:**
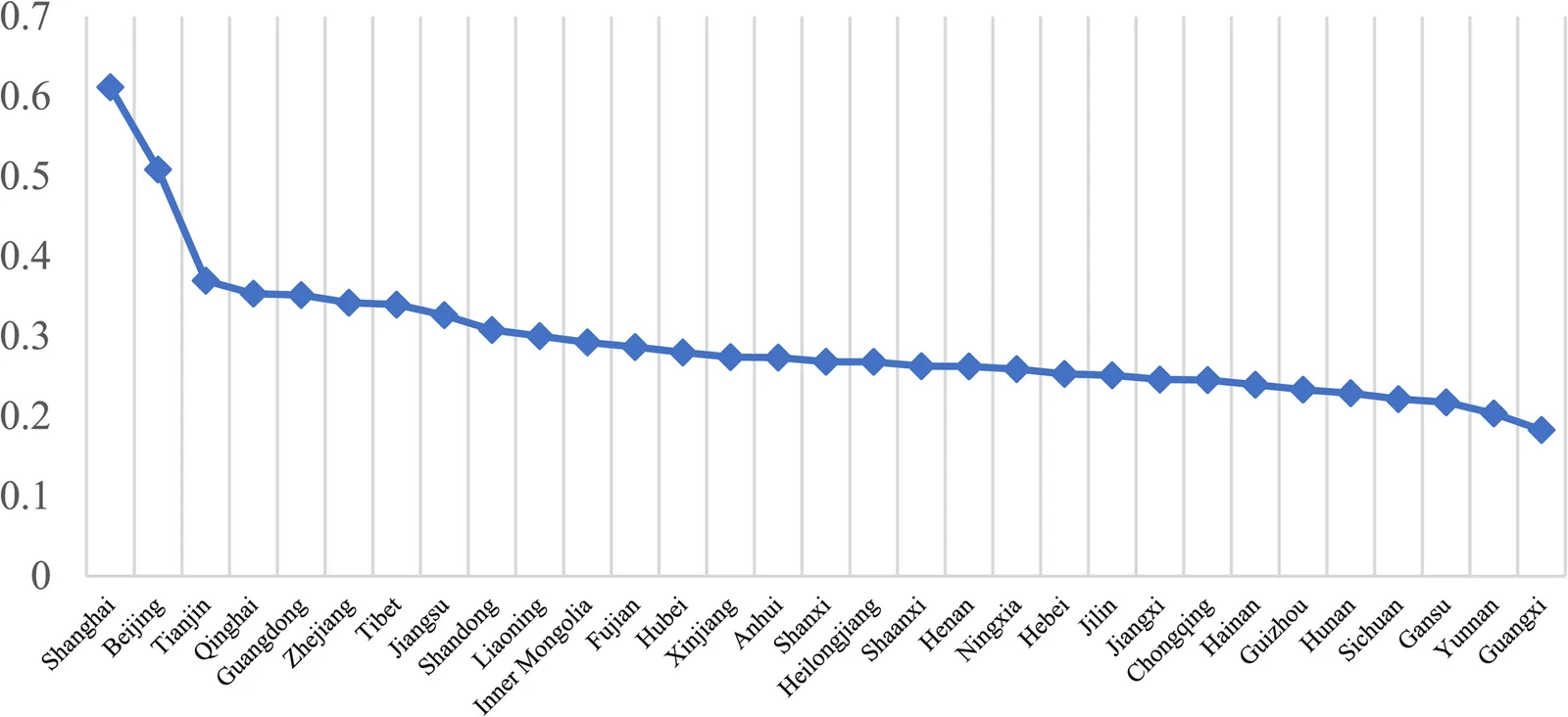
Average public health emergency response capacity by province, 2013–2021

By analyzing the spatial distribution of the regional emergency response capacity for public health emergencies in each province from 2013 to 2021, as shown in Fig. 2, it is found that the regional emergency response capacity in the eastern coastal region is higher than that in the inland region, but this difference is decreasing year by year. In addition, the emergency response capacity for public health emergencies in each province over the past years was ranked based on their average values, and the ranking results are shown in Fig. 3. The average emergency response capacity for public health emergencies across China’s 31 provinces from 2013 to 2021 is 0.292. Among these, 11 provinces, including Shanghai and Beijing, have an emergency response capacity higher than the national average, while the remaining 20 provinces fall below the national average. There is a big difference in the emergency response capacity of public health emergencies among provinces, among which Shanghai has the highest, with a relative proximity of 0.61, and Guangxi has the lowest, with a relative proximity of 0.18, and a difference of about 0.43 with Shanghai, which indicates that there are obvious regional differences in the emergency response capacity of public health emergencies in China, and there is an unbalanced development situation.

#### Comparison of regional emergency response capabilities from a three-stage perspective

**Fig. 4:**
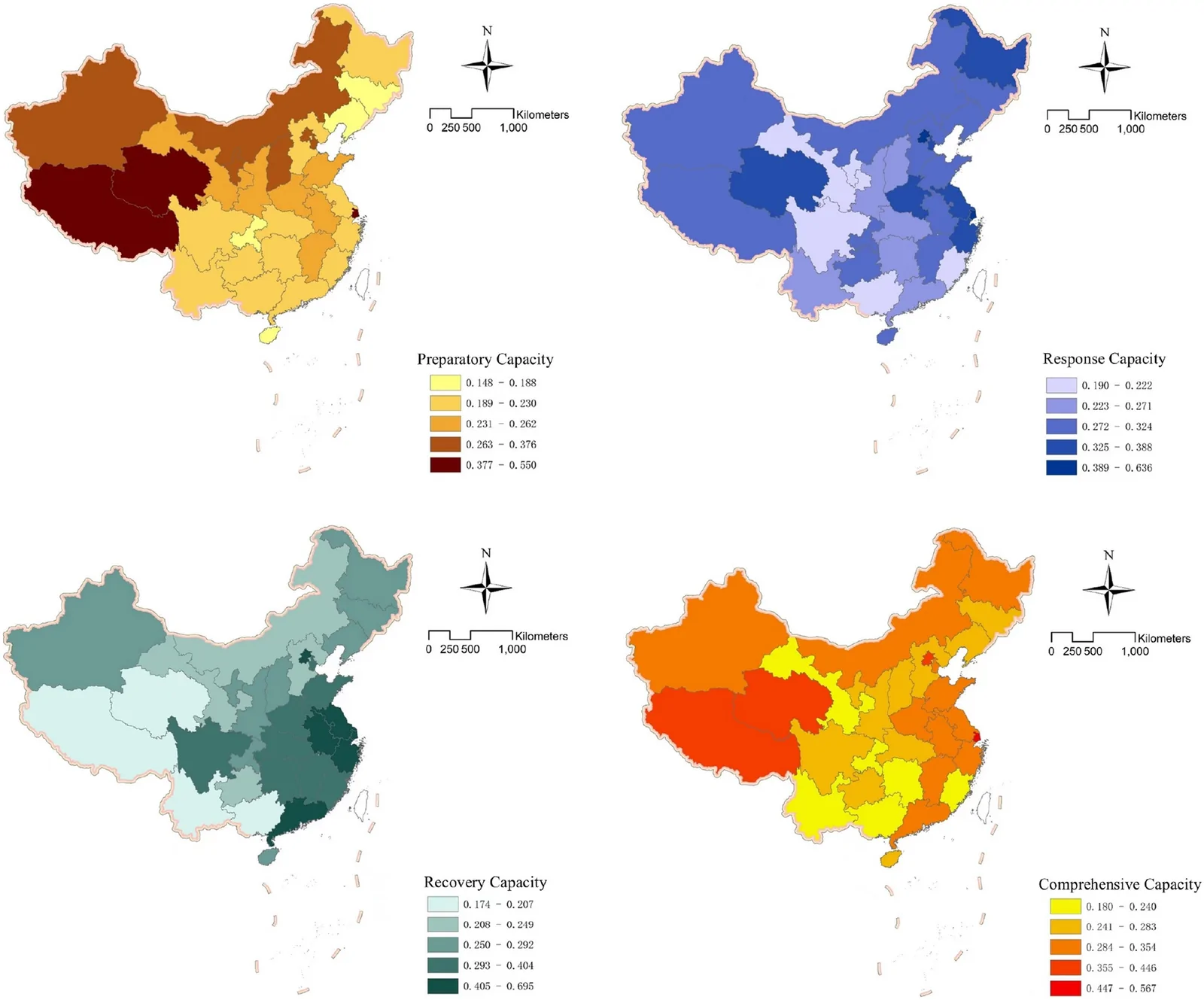
Spatial distribution map of Level-1 indicators for regional public health emergency response capacity by province, 2021

Further analyze the evaluation results of the emergency response capacity of each level 1 indicator, take 2021 as an example, analyze and compare the emergency response capacity of each province under the three level 1 indicators of pre-preparation, in-process response and post-recovery, and get the evaluation results of the level 1 indicators of the emergency response capacity of public health emergencies in each province in 2021, as shown in Table 3, and to compare the differences in the spatial distribution of the data in a clear way, the data are visualized as Fig. 4.

**Table 3:** Provincial variations in public health emergency response capacity: dimensional analysis and comprehensive relative proximity scores (2021)

Province	Pre-preparation capacity	In-process response capacity	Post-recovery capacity	Comprehensive emergency response capacity
Beijing	0.288	0.528	0.558	0.446
Tianjin	0.303	0.345	0.356	0.319
Hebei	0.222	0.280	0.249	0.252
Shanxi	0.297	0.239	0.273	0.261
Inner Mongolia	0.312	0.308	0.242	0.298
Liaoning	0.165	0.314	0.277	0.262
Jilin	0.148	0.301	0.274	0.256
Heilongjiang	0.210	0.357	0.271	0.301
Shanghai	0.468	0.636	0.695	0.567
Jiangsu	0.201	0.350	0.476	0.332
Zhejiang	0.214	0.333	0.495	0.342
Anhui	0.253	0.285	0.481	0.320
Fujian	0.215	0.222	0.326	0.240
Jiangxi	0.262	0.308	0.336	0.301
Shandong	0.248	0.324	0.372	0.306
Henan	0.244	0.388	0.376	0.354
Hubei	0.230	0.271	0.404	0.283
Hunan	0.211	0.238	0.329	0.239
Guangdong	0.218	0.264	0.495	0.308
Guangxi	0.205	0.190	0.174	0.180
Hainan	0.188	0.317	0.274	0.279
Chongqing	0.183	0.287	0.261	0.240
Sichuan	0.218	0.211	0.369	0.259
Guizhou	0.212	0.318	0.229	0.267
Yunnan	0.224	0.256	0.180	0.225
Tibet	0.550	0.295	0.189	0.393
Shaanxi	0.255	0.246	0.292	0.259
Gansu	0.245	0.218	0.247	0.229
Qinghai	0.470	0.368	0.207	0.379
Ningxia	0.308	0.195	0.287	0.260
Xinjiang	0.376	0.286	0.270	0.326

As shown in Fig. 4 and Table 3, significant spatial and stage-wise disparities are observed in the evaluation of regional emergency response capacity for public health emergencies. The pre-preparation stage exhibits the lowest overall capacity, with a provincial average of 0.26. Spatially, the western region demonstrates higher capacity in this stage than the eastern and central regions. Although Tibet, Qinghai, and Shanghai lead in this dimension, most provinces show weaker performance in pre-preparation compared to subsequent stages. In contrast, the in-process response stage demonstrates a higher average score of 0.306, with both eastern and western regions performing better than the central region. Meanwhile, the post-recovery stage shows an average of 0.331, with the eastern region outperforming both the central and western regions.

Generally speaking, the emergency response capacity of most provinces in the pre-preparation stage is at a lower level than that in the in-process response and post-recovery stages. Most provinces pay more attention to the response and recovery phases and invest less in the preparation phase. And the conclusion of this paper is in line with the “comet” structure of emergency management [43], in which rescue and disposal are in the nucleus of the comet, while prevention and preparedness, monitoring and early warning are in the tail of the comet. The proposed health and wellness policy of “prevention priority” is improving the unbalanced structure of the entire process of public health emergency management, and there is still much room for future growth in the capacity to prepare for emergencies beforehand.

#### Analysis of the structural differences in public health emergency response capabilities among provinces in the eastern, central and western regions

**Fig. 5:**
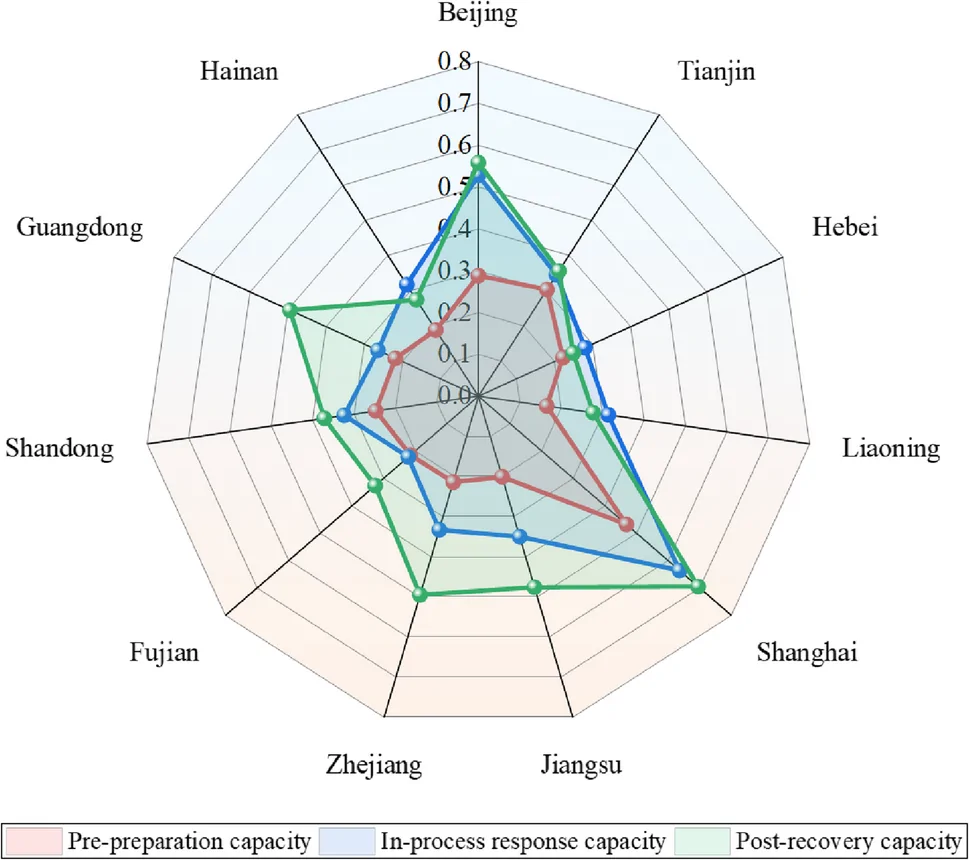
Distribution of public health emergency response capacity in the eastern region, 2021

**Fig. 6:**
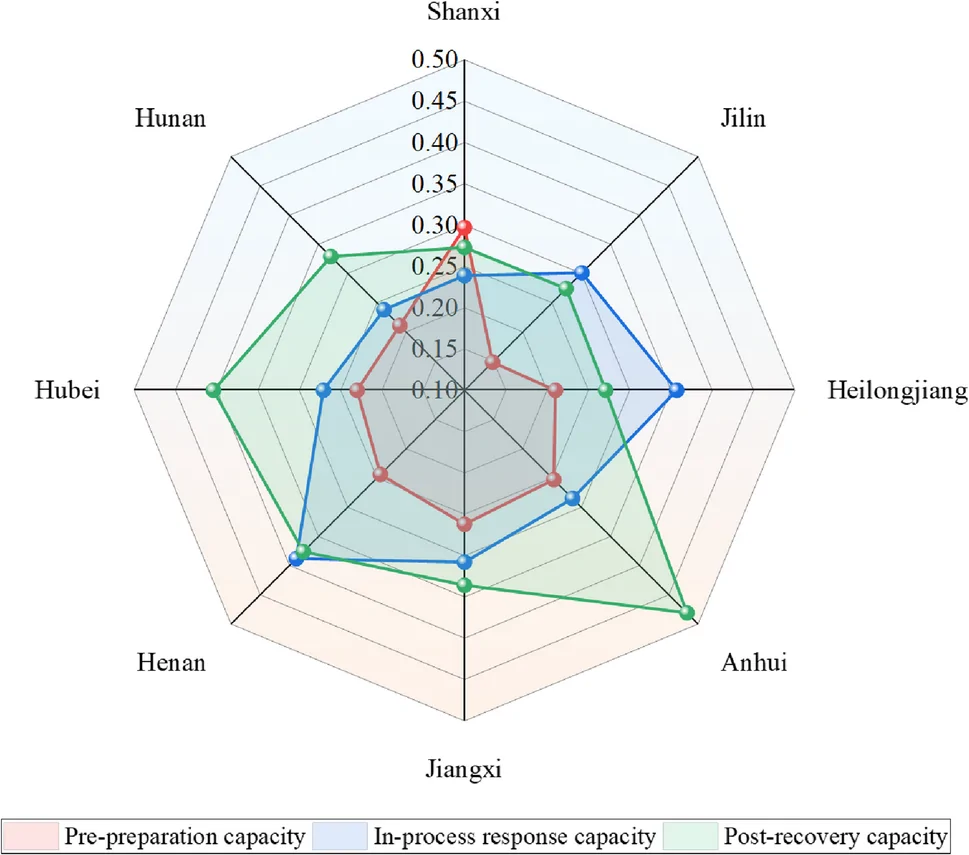
Distribution of public health emergency response capacity in the central region, 2021

**Fig. 7:**
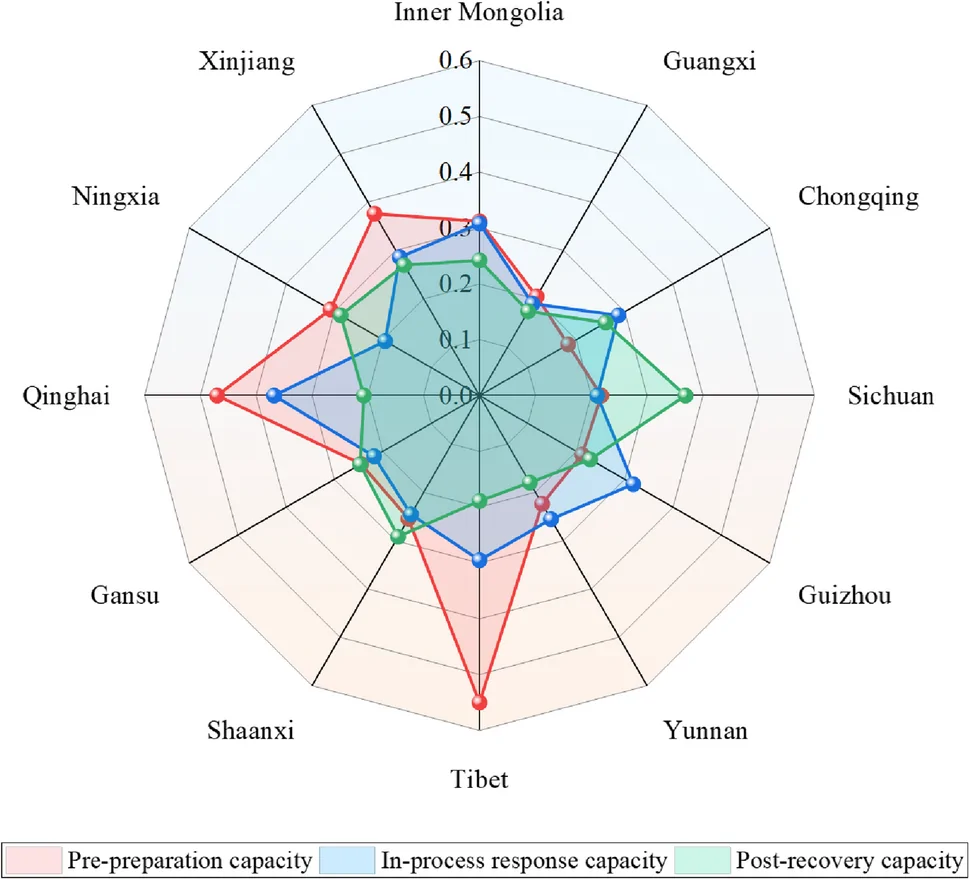
Distribution of public health emergency response capacity in the western region, 2021

To further analyze the difference in regional emergency response capacity for public health emergencies, the 31 provinces were divided into eastern, central and western regions^1^. And radar charts of the results of the evaluation of the emergency response capacity for public health emergencies in the eastern, central and western regions in 2021 were plotted in Figs. 5, 6 and 7, respectively.

- (1) Eastern region: The pre-preparation capacity of all provinces in the eastern region is lower than the in-process response capacity and post-recovery capacity, with Shanghai having the highest level of emergency response capacity in all three dimensions, as shown in Fig. 5.
- (2) Central region: Among the eight central Chinese provinces, capacities in pre-preparation, in-process response, and post-recovery show relatively small variations. Most provinces exhibit lower pre-preparation capabilities compared to response and recovery, with Shanxi being an exception—recording the highest pre-preparation score of 0.286. Henan achieved the highest in-process response capacity of 0.433, attributable to its top national Internet penetration rate of 91.9%, which facilitates rapid public information dissemination, as well as superior concentration of hospitals and healthcare personnel. Anhui led in post-recovery capacity of 0.43, supported by strong scientific investment—with over 5% of fiscal expenditure allocated to science and technology—and extensive technical consultations and policy recommendations of 1,235 instances in 2021. These findings highlight Internet accessibility and expert-involved policy consultation as key factors enhancing response and recovery capabilities in public health emergencies.
- (3) Western region: In the western region, significant disparities exist in public health emergency management capacities across the 12 provinces. Tibet, Qinghai, Ningxia, and Xinjiang exhibit stronger pre-preparation than response and recovery capabilities, reflecting a governance shift towards prevention. Tibet leads in pre-preparation, supported by its “prevention-first” policy and extensive CDC institutional coverage of 82 institutions in 2021. However, as Class A and B notifiable infectious-disease hotspots are concentrated in western regions such as Xinjiang and Tibet, this poses a severe test to their own in-process response and post-recovery capacities. Qinghai has the highest in-process response capacity of 0.368, though its post-recovery performance is weak of 0.207. Sichuan demonstrates the strongest post-recovery capacity of 0.369, likely due to its experience with frequent natural disasters, which has strengthened its recovery and summary evaluation processes, despite lower capabilities in pre-preparation and response.

In pre-preparation, despite Beijing’s advantages in medical resources and technological application, the city shows relative inadequacies in preventive health beds and health education and publicity, constraining its preventive and early intervention capacities. During in-process response, both Beijing and Shanghai benefit from high transport network density of 2.14 and 1.49, respectively, facilitating rapid coordination of medical resources, personnel deployment, and interagency collaboration, further supported by high population density and concentrated medical resources. In post-recovery, Beijing and Shanghai exceed the national average in rehabilitation and psychiatric beds, while higher science and technology expenditure in Beijing, Shanghai, Zhejiang, and Guangdong enhances their capacity for developing effective recovery solutions and medical technologies, thus their ability to recover from the aftermath of such emergencies is stronger.

### Longitudinal comparison of historical changes

**Fig. 8:**
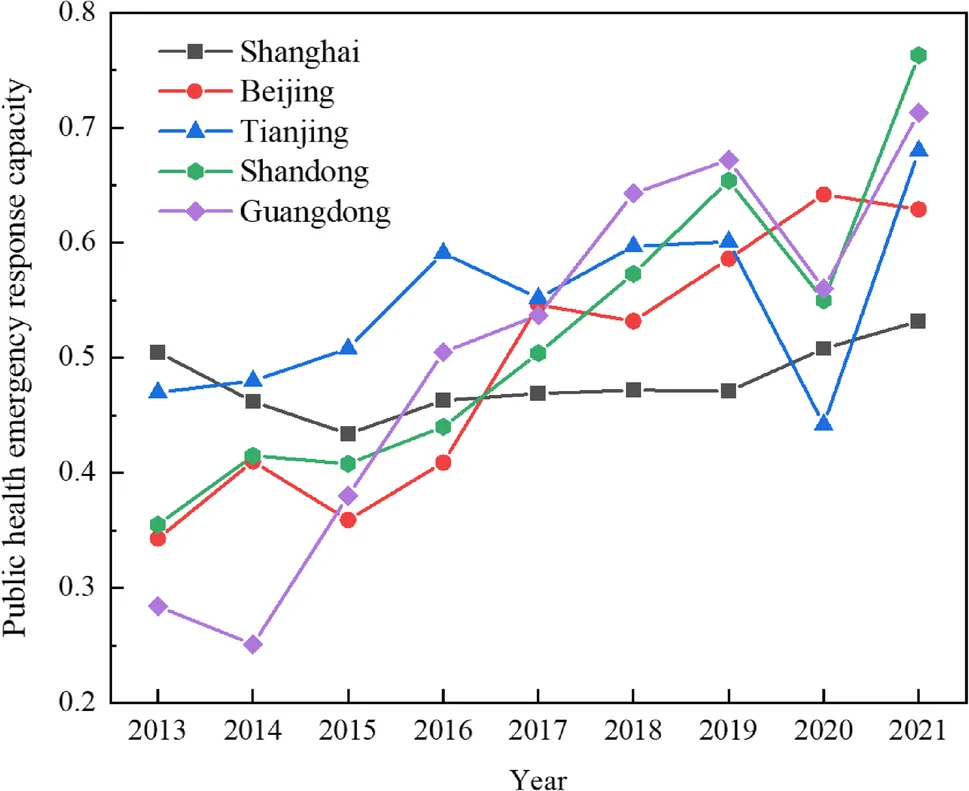
Evaluation of public health emergency response capacity by province in the eastern region, 2013–2021

**Fig. 9:**
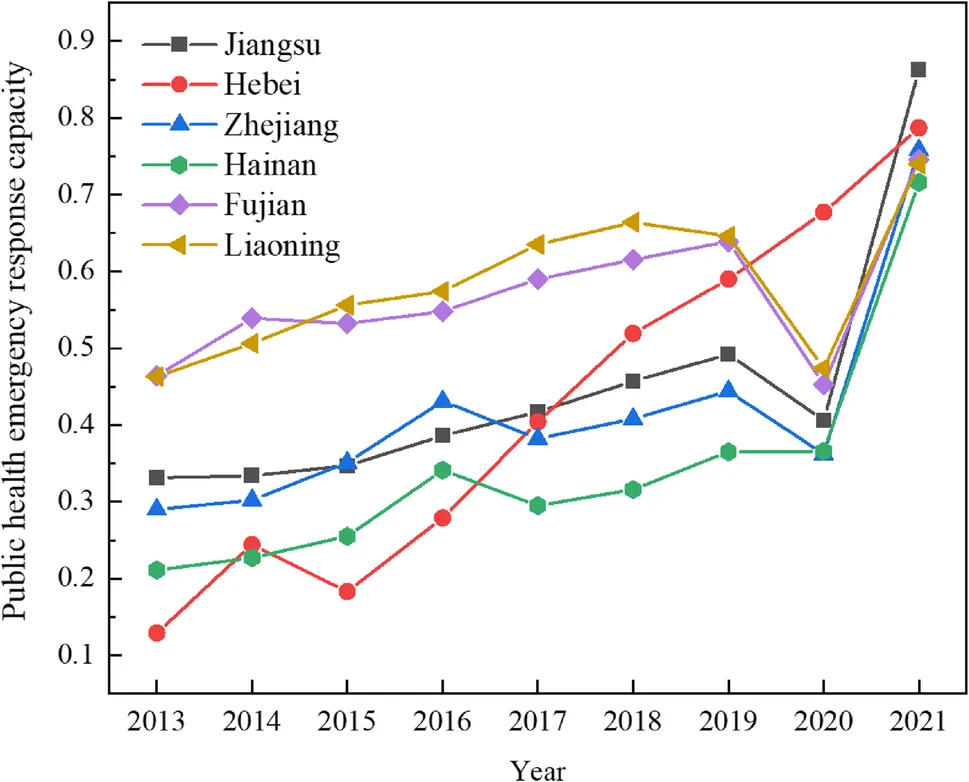
Evaluation of public health emergency response capacity by province in the eastern region, 2013–2021 (continued)

**Fig. 10:**
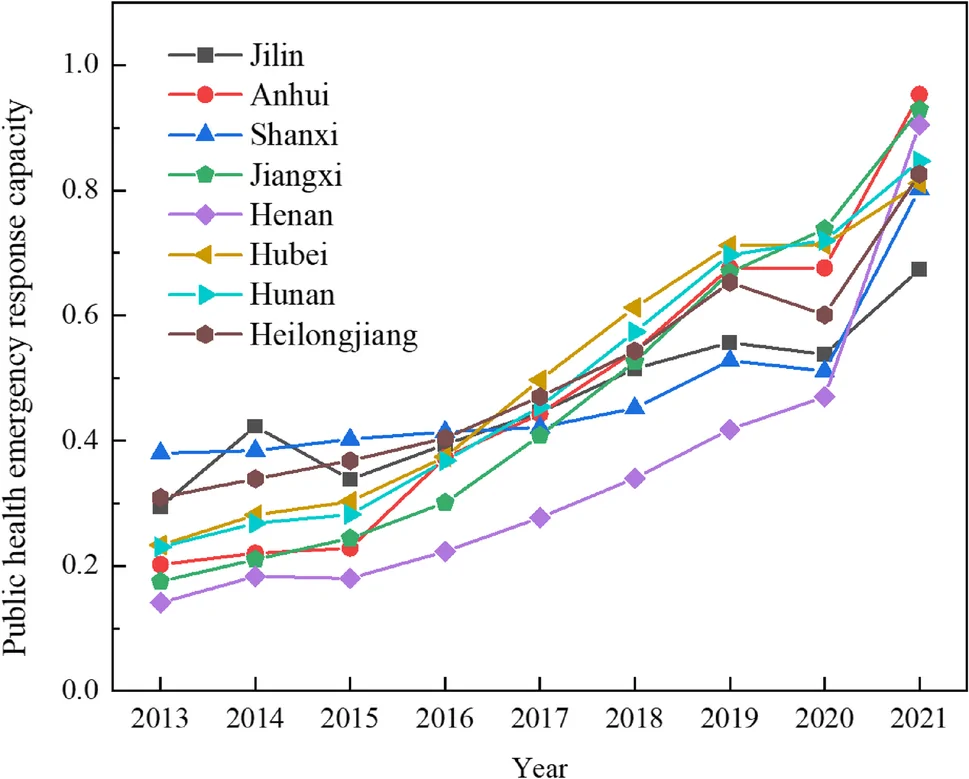
Evaluation of public health emergency response capacity by province in the central region, 2013–2021

**Fig. 11:**
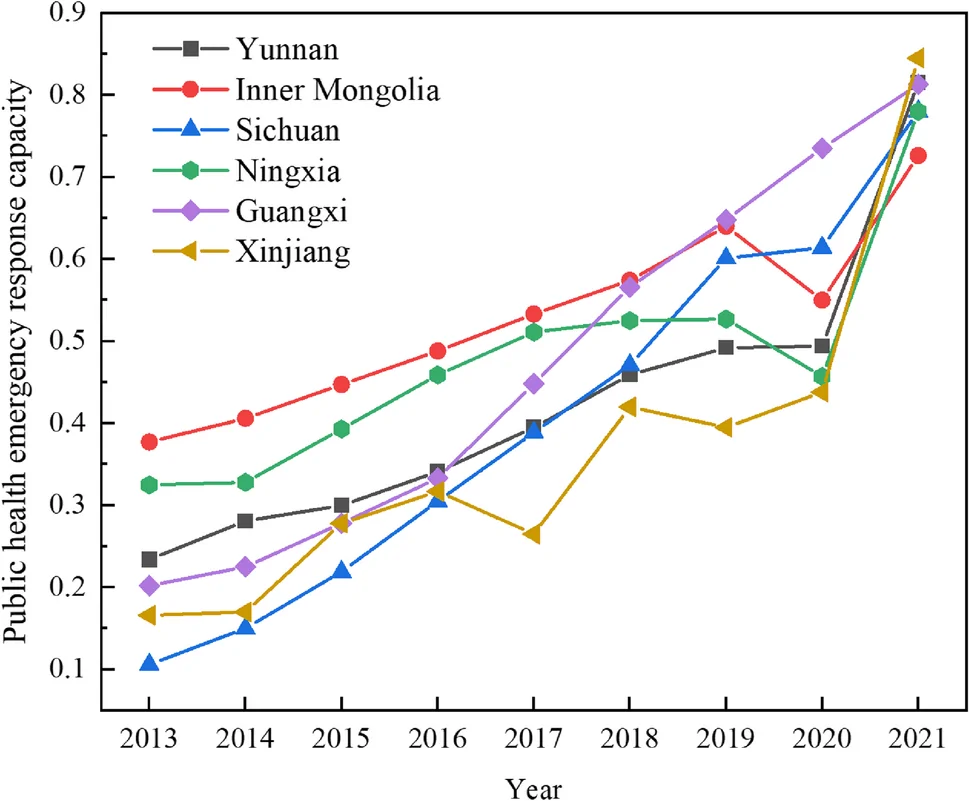
Evaluation of public health emergency response capacity by province in the western region, 2013–2021

**Fig. 12:**
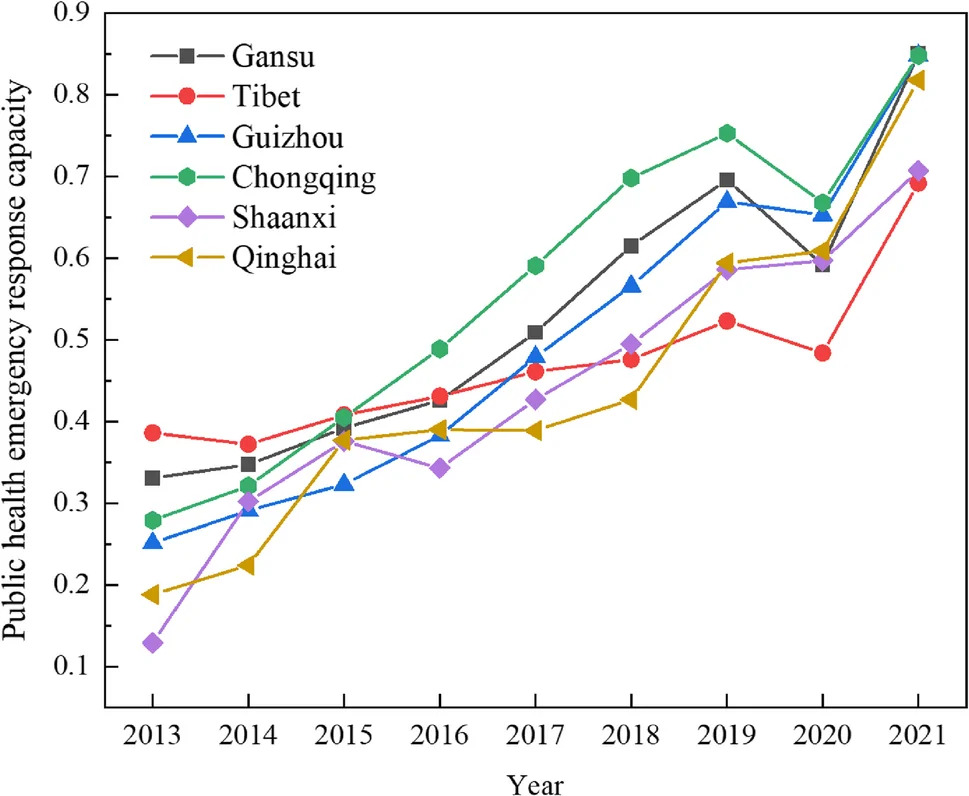
Evaluation of public health emergency response capacity by province in the western region, 2013–2021 (continued)

A longitudinal evaluation of the emergency response capacity for public health emergencies was conducted in the eastern, central, and western regions, respectively, using time-series data on the relative proximity of each province’s emergency response capacity from 2013 to 2021. The relative proximity results for the emergency response capacity of public-health emergencies across provinces in the eastern, central and western regions from 2013 to 2021 are presented in Table C1- C3 of Appendix C, respectively, and illustrated in Figs. 8, 9, 10, 11 and 12.

Eastern China generally showed an upward trend in public health emergency response capacity from 2013 to 2021. As international metropolises, Beijing and Shanghai demonstrated relatively stable and balanced growth owing to their mature management mechanisms and efficient resource deployment. However, most coastal provinces (Tianjin, Shandong, Guangdong, Jiangsu, Zhejiang, Fujian and Liaoning) experienced a sharp decline in capacity in 2020 due to the sudden outbreak of COVID-19, which overwhelmed existing resources and response strategies. Subsequently, reforms in public health emergency management systems led to a rapid rebound in 2021, with Fujian showing the most noticeable improvement. The pandemic-triggered reforms, namely enhanced healthcare infrastructure, interagency coordination and technological surveillance, significantly strengthened regional response capabilities. It is anticipated that the emergency response capacity across eastern provinces will continue to grow in the coming years.

In the central region, the upward trend of the emergency response capacity for public health emergencies in each province from 2013 to 2021 is more obvious, as shown in Table C2 of Appendix C and Fig. 10. From an overall perspective, except for 2015 and 2020, when the emergency response capacity of some provinces declined, all other years showed an increase.

Western China also exhibited an overall upward trend in public health emergency response capacity from 2013 to 2021. Xinjiang demonstrated a distinct pattern, with accelerated improvement in 2018 and 2021 following declines in 2017 and 2019. This rebound is largely attributed to strengthened enforcement of *the Law of the People’s Republic of China on Prevention and Treatment of Infectious Diseases*^2^, and subsequent local reforms, which enhanced infectious disease management and facilitated comprehensive emergency response capacity building.

### Analysis of factors affecting regional emergency response capacity for public health emergencies

#### Selection of regional emergency response capacity factors for public health emergencies

Referring to the existing literature, seven indicators with regional characteristics, including economic development level [44], health consumption capacity [13], education level [45], informatization level [46], transport network density [25], disastrous factors [47] and epidemic intensity of infectious disease [48], were selected to explore and analyze the influencing factors of public health emergency response capacity.

In this paper, gross domestic product per capita (PGDP) is used to measure the level of regional economic development, and total health expenditure per capita (PTHE) reflects health consumption capacity [13], the level of education (ED) is quantified by the average number of students enrolled in higher education per 100,000 population, the Internet penetration rate (NET) reflects the level of informatization, the density of transport networks (TRANS) characterizes the connectivity of the physical infrastructure, and the total number of disastrous factors (DISASTER) measures the frequency of geological disasters, earthquake disasters and environmental emergencies, in a certain region [47]. The incidence rate of class A and B notifiable infectious diseases (MORBIDITY) is a proxy for the epidemic intensity of infectious disease. Among them, Transport network density and Disastrous factors belong to the physical space, Economic development level, Health consumption capacity, Education level, Epidemic intensity of infectious disease belong to the social space. Informatization level belongs to the information space, so the influencing factors cover the triad of physical-social-information space, which is more comprehensive.

#### Analysis of influencing factors based on the SHAP value method

**Fig. 13:**
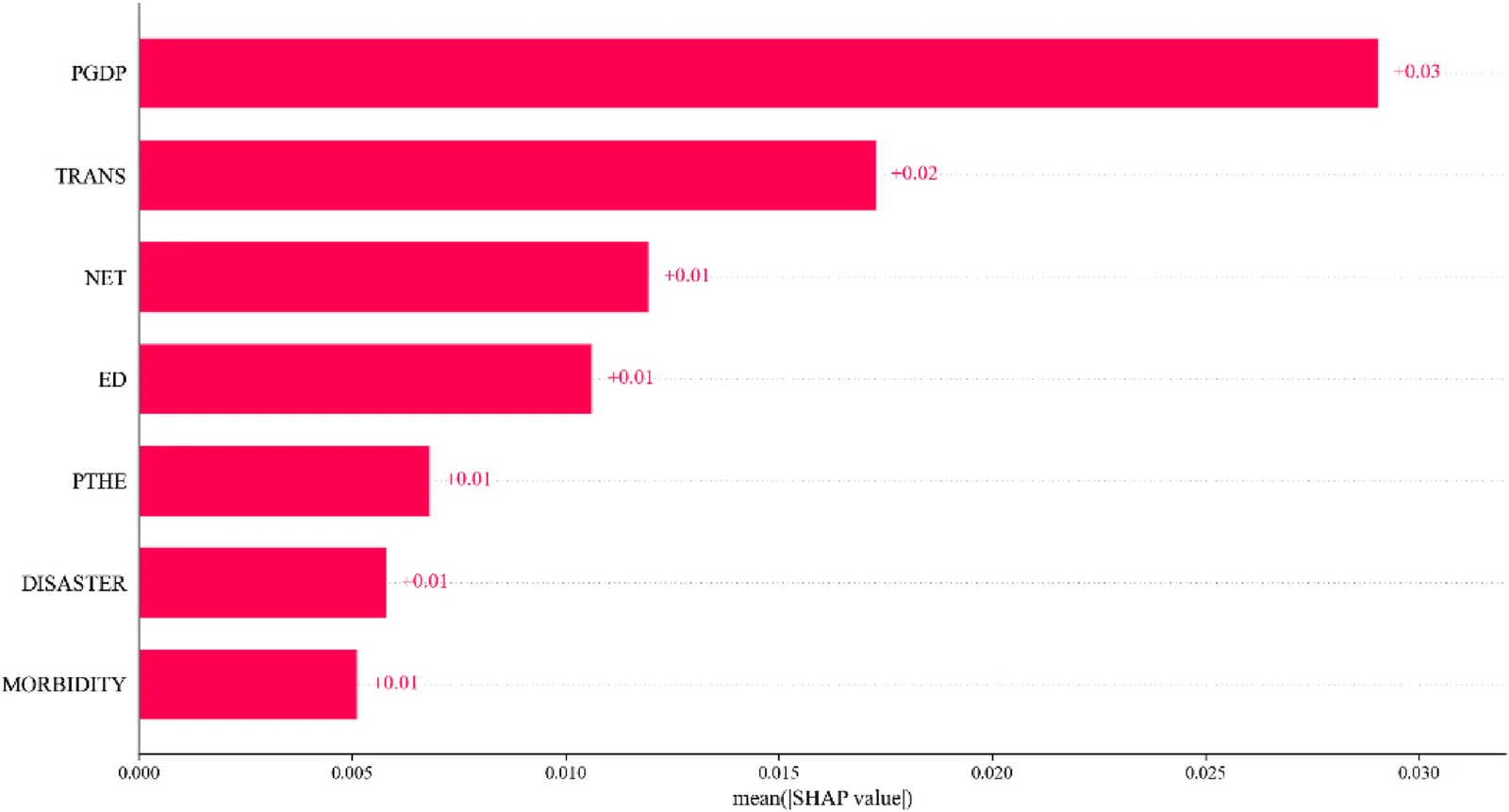
Global feature importance plot based on SHAP values

**Fig. 14:**
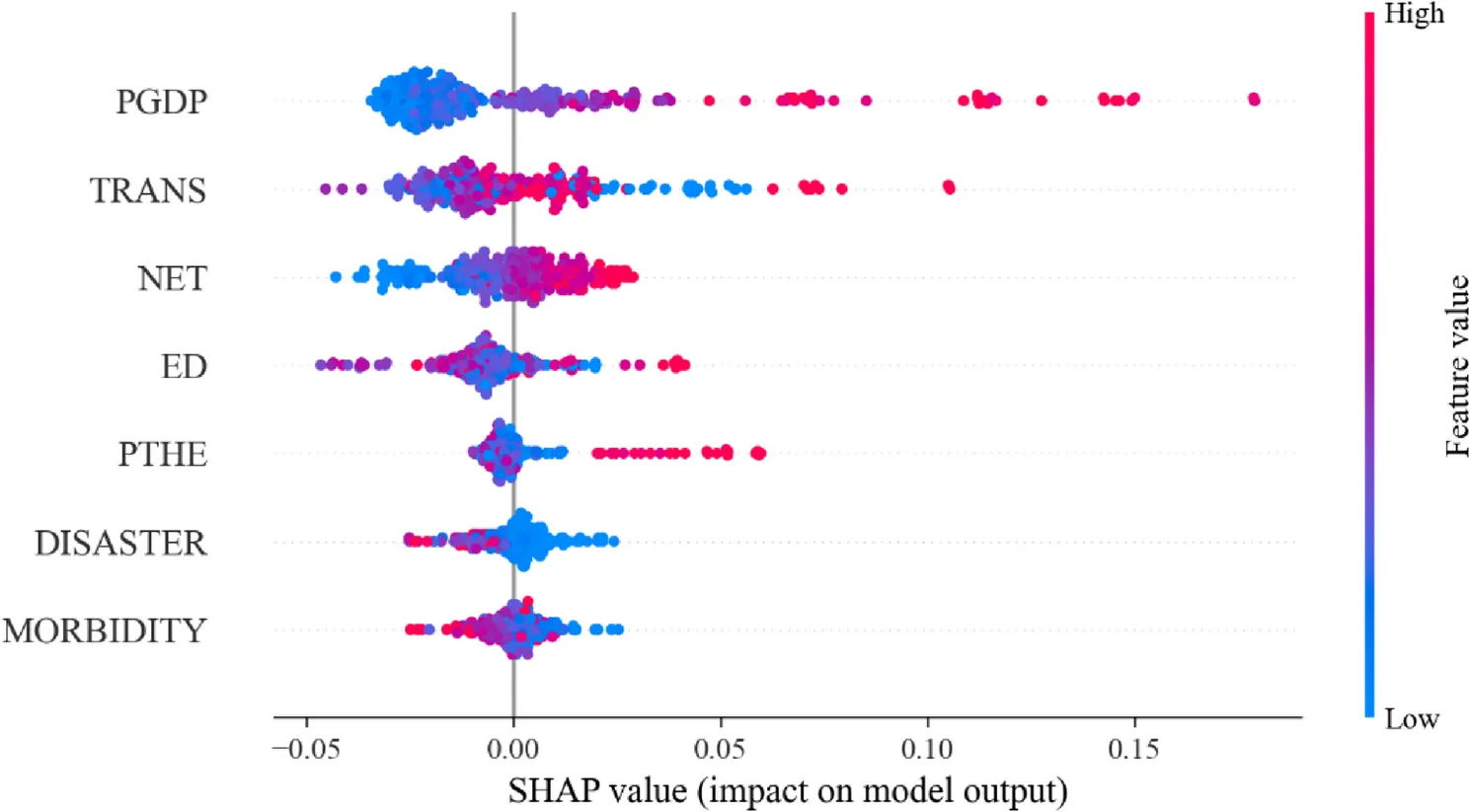
Beeswarm plot based on SHAP values

The SHAP model was used to assess the importance of the above indicators, such as economic development level, education level and other regional characteristics. The importance of each factor to the emergency response capacity of public health emergencies is highlighted by SHAP feature importance plot and beeswarm plot, and the results are shown in Figs. 13 and 14. Figure 13 shows that the most important factors affecting the regional emergency response capacity of public health emergencies are the Economic development level and the Transport network density.

Figure 14 shows that the higher the PGDP and TRANS, the higher the regional public health emergency response capacity. Regions with a high level of economic development usually have more sufficient financial resources to invest in public health infrastructure construction, training of healthcare personnel and scientific research and innovation, which plays a key role in the improvement of regional emergency response capacity for public health emergencies. The dense and efficient transport network is the vein of material flow, which is the embodiment of emergency response speed. Emergency logistics, as a key part of rescue operations, plays an important role in the response to public health emergencies [49]. The correlation between the two and the regional public health emergency response capacity is consistent with the finding that regions with a high level of economic development and a dense transport network, such as Beijing and Shanghai, have a higher public health emergency response capacity.

In addition, the NET and PTHE also contribute positively to the public health emergency response capacity. With the advancement of digitalization, the Internet and smart technologies have risen to become the core driving force for strengthening the emergency response capacity of public health emergencies. Through intelligent and networked means, it is possible to integrate isolated and fragmented information and material resources, and to build an efficient, coordinated and responsive emergency decision-making hub [26]. The increase in PTHE reflects the importance that regional citizens attach to health and their spending power [13], which is directly related to the abundance of medical resources, and therefore has a positive impact on the enhancement of the regional capacity to respond to public health emergencies.

The DISASTER, as well as the MORBIDITY, shows an inverse trend to the public health emergency response capacity. The various types of emergencies have a “ripple effect”, affecting and transforming each other, and the occurrence of natural disasters and calamitous accidents may trigger the emergence of public health events [50]. Regions with a high incidence of infectious diseases and other public health events often face greater public health challenges, which can easily expose the loopholes and deficiencies in the public health emergency system, and the vulnerability of the system is further magnified.

For the indicator of ED, i.e., the average number of students enrolled in higher education per 100,000 population, there is no obvious positive gain or negative reduction effect on the emergency response capacity of public health emergencies due to the relatively uniform distribution of positive and negative sample points on the horizontal axis. The allocation and utilization of higher education resources need to be more accurately aligned with the needs of public health emergencies, such as more specific public health education, training and practice, in order to be transformed into the actual power of emergency capacity improvement.

## Conclusions and recommendations

### Key findings

The study reveals four critical insights regarding regional public health emergency response capabilities in China.

- (1) Pronounced regional disparities exist, with eastern provinces demonstrating superior emergency response capabilities due to stronger economic foundations and more developed infrastructure, while certain western regions exhibit strengths in preventive measures.
- (2) An imbalanced “comet-shaped” structure characterizes the emergency management system, where pre-preparation significantly trails in-process response and post-recovery capacities; however, following the 2018 establishment of the Ministry of Emergency Management, China’s public-security governance model has steadily shifted toward ex-ante prevention, narrowing the preparedness gap over time.
- (3) Despite this imbalance, provincial emergency response capacity generally followed a consistent upward trajectory from 2013 to 2021, with most provinces rebounding to record levels after the 2020 COVID-19 shock.
- (4) Economic development level and transportation network density emerge as pivotal determinants of emergency capacity, whereas high infectious disease incidence and frequent disasters negatively impact response effectiveness.

### Policy recommendations

To enhance China’s public health emergency management system, the following evidence-based policy measures are proposed:

- Firstly, preparedness systems require strengthening through targeted resource allocation. Fiscal investments should prioritize disease control centers and primary healthcare institutions, particularly in remote areas, with attention to optimizing the geographic distribution of critical resources such as hospital beds and medical personnel. Concurrently, public awareness campaigns leveraging digital platforms can improve risk communication and community resilience.
- Secondly, regional disparities must be addressed through coordinated development strategies. Establishing paired assistance mechanisms between eastern and western provinces would facilitate knowledge transfer and technological cooperation, including the adoption of 5G-enabled telemedicine and big data surveillance systems. Infrastructure development in central and western regions, particularly transportation networks and digital health platforms, should be prioritized to ensure equitable emergency response capabilities nationwide.
- Thirdly, a tri-space governance framework integrating physical, social, and informational dimensions should be implemented. This includes optimizing the spatial distribution of medical facilities, institutionalizing cross-departmental emergency drills, and developing a national big data platform for comprehensive epidemic surveillance and resource management.
- Finally, economic and technological enablers should be leveraged. Incorporating public health emergency capacity into local government performance evaluations can incentivize investments in vulnerable regions. Advanced technologies such as artificial intelligence and drone logistics should be deployed to enhance emergency supply chains, particularly in areas with challenging terrain.

### Limitations and further works

The study relies on publicly available data from national statistical sources, which may have limitations in terms of granularity and timeliness. Some provinces might have more detailed data available at the local level, which could provide a more nuanced understanding of their emergency response capabilities. Additionally, the current study uses a cross-sectional approach to analyze emergency response capabilities over a period of time. While this provides a snapshot of overall trends, it does not capture the dynamic changes in capabilities at different stages in a detailed manner. Future research could benefit from using fixed effects or random effects models to analyze the dynamic changes in these capabilities over time.

## Supplementary Information

Supplementary Material 1: Appendix A introduces specific methods, including game combination weighting, the CRITIC method, entropy weighting, AHP, and TOPSIS.

Supplementary Material 2: Appendix B provides weighting information for the evaluation indicators.

Supplementary Material 3: Appendix C illustrates relative proximity of public health emergency response capacity by province in the three regions, 2013-2021.

## Data Availability

Data will be made available on request.

## Abbreviations

TOPSIS: Technique for Order Preference by Similarity to Ideal Solution
SARS: Severe Acute Respiratory Syndrome
SHAP: Shapley Additive Explanations
NHSPI: National Health Security Preparedness Index
CDC: Center for Disease Control and Prevention
AHP: Analytic Hierarchy Process
CRITIC: Criteria Importance Through Intercriteria Correlation
DEA: Data Envelopment Analysis
PGDP: Gross domestic product per capita
PTHE: Total health expenditure per capita
ED: Education
NET: Internet penetration rate
TRANS: The density of transport networks
DISASTER: The total number of disastrous factors

## Footnotes

1 The division of eastern, central, and western regions is based on *China’s Hygiene and Health Statistical Yearbook* (http://www.nhc.gov.cn/mohwsbwstjxxzx/tjtjnj/tjsj_list.shtml). The eastern region includes 11 provinces (municipalities directly under the central government), including Beijing, Tianjin, Hebei, Liaoning, Shanghai, Jiangsu, Zhejiang, Fujian, Shandong, Guangdong and Hainan; the central region includes 8 provinces, including Heilongjiang, Jilin, Shanxi, Anhui, Jiangxi, Henan, Hubei and Hunan; and the western region includes 12 provinces (autonomous regions and municipalities directly under the central government), including Inner Mongolia, Guangxi, Chongqing, Sichuan, Guizhou, Yunnan, Tibet, Shaanxi, Gansu, Qinghai, Ningxia and Xinjiang.

2
http://npc.people.com.cn/n1/2018/0830/c14576-30261241.html.
