# Supplementary material for: Indicator system for regional public health emergency response capacity based on ternary space theory: construction and empirical study in China
Source: BMC Public Health. 2026 Aug 1;26:3330. doi: 10.1186/s12889-026-28703-4 (PMC13753278; doi:10.1186/s12889-026-28703-4)
Supplement: Supplementary file 1 [file 12889_2026_28703_MOESM1_ESM.docx]

**Appendix A. Detailed Methodology**

**1. Game Theory-Based Combination Weighting Method**

Game theory is a mathematical theory and method for studying competitive things in the field of operations research. The basic idea is to find an equilibrium point between subjective and objective weights, i.e., to compare and coordinate different weights to obtain the optimal combination of weights that maximizes common interests^[1]^. This study combines AHP (analytic hierarchy process)^[2]^, CRITIC (criteria importance through inter-criteria correlation)^[3]^, and entropy weight method^[4]^ to solve the comprehensive weights of indicators, and then applies the game theory combination weighting method to obtain the final attribute weight information. It not only reflects the preference of the decision maker, but also takes into account the objective data related to the assessment object, hence enhancing the reliability of the evaluation result. It can consider both the subjective judgment of the decision maker and the objective measurements of the object being evaluated, resulting in the optimal combination of the three assessment methods. The specific steps are as follows.

***Step 1*:** Data Normalization and Standardization. Prior to analytical computations, the raw data underwent normalization and dimensionality reduction processes to mitigate the influence of varying measurement scales and directional inconsistencies. The present study employed a stepwise transformation methodology to process the initial dataset, subsequently generating a standardized matrix suitable for comparative analysis and quantitative evaluation.

For benefit indicators:

$$y_{ij}=\left(r_{ij}-r_{\min}\right)/\left(r_{\max}-r_{\min}\right)$$

For cost indicators:

$$y_{ij}=\left(r_{\max}-r_{ij}\right)/\left(r_{\max}-r_{\min}\right)$$

where$r_{\max},r_{\min}$ are the maximum and minimum values in index *j* respectively.

Since there is still data that is zero after the step transformation method of processing, the standardized matrix continues to be non-negatively shifted^[5]^, which translates the coordinates:

$$y_{ij}=y_{ij}+d, d=0.0001$$

***Step 2*** Build the basic weight vector set, where $W_{q}$ is the weight set determined by the *q-*th assignment method, *n* is the number of indicators, and *p* is the number of weighting methods. Since this paper mainly combines AHP, CRITIC and entropy weight methods to solve the comprehensive weight of indicators (see the appendix for the specific calculation process), therefore, *p* is taken as 3. The weight vectors composed by the three methods are linearly combined:

$$W=\alpha_{1}w_{1}^{T}+\alpha_{2}w_{2}^{T}+\alpha_{3}w_{3}^{T}$$

where$\alpha_{1},\alpha_{2},\alpha_{3}$ are the weight coefficients of AHP, CRITIC, and entropy weight methods respectively.

***Step 3*** Based on the idea of the game agglomeration model, to minimize the deviation, optimizing the three linear combination coefficients, the optimal weights can be derived, from which the objective function is determined:

$$\min\left\|W-w_{p}^{T}\right\|_{2},p=1,2,3$$

*W* is the set of vectors for summing the combined weights that satisfy the minimum Euclidean distance between the set of target vectors and the set of original vectors.

***Step 4*** Based on the differential character of the matrix, Eq. can be transformed into a system of linear equations with an optimized first-order derivative condition:

$$\left(\begin{matrix} w_{1}\times w_{1}^{T} & w_{1}\times w_{2}^{T} & w_{1}\times w_{3}^{T} \\ w_{2}\times w_{1}^{T} & w_{2}\times w_{2}^{T} & w_{2}\times w_{3}^{T} \\ w_{3}\times w_{1}^{T} & w_{3}\times w_{2}^{T} & w_{3}\times w_{3}^{T} \end{matrix}\right)\left(\begin{matrix} \alpha_{1} \\ \alpha_{2} \\ \alpha_{3} \end{matrix}\right)=\left(\begin{matrix} w_{1}\times w_{1}^{T} \\ w_{2}\times w_{2}^{T} \\ w_{3}\times w_{3}^{T} \end{matrix}\right)$$

***Step 5*** Normalize the optimized linear combination coefficients $\alpha_{1}^{*},\alpha_{2}^{*},\alpha_{3}^{*}$ :

$$\alpha'_{p}=\frac{\alpha_{p}^{*}}{\sum_{p=1}^{3} \alpha_{p}^{*}}, p=1,2,3$$

***Step 6*** The final combination weight *W* is:

$$W=\alpha'_{1}w_{1}^{T}+\alpha'_{2}w_{2}^{T}+\alpha'_{3}w_{3}^{T}$$

**2. CRITIC methodology**

The CRITIC method is an objective weighting method that takes into account data variability and correlation between data. The standard deviation is used to indicate the variability of the indicator; the larger the standard deviation, the greater the difference in the value of the indicator, the more information it reflects, and the higher the weight; the correlation coefficient is used to indicate the correlation between indicators; the larger the correlation coefficient between the indicators, the smaller the conflict, the smaller the amount of information contained in the indicator, and the lower the weight^[3]^. The specific calculation process is as follows:

1) Calculation of indicator variability. The standard deviation of the *jth* indicator is denoted by$\sigma_{j}$ and the corresponding formula is:

Where $x_{i}$ is the *ith* attribute value of indicator *j*, and$\overline{x}$ is the arithmetic mean of$x_{i}$.

2) Calculation of indicator correlation.

$$R_{j}=\sum_{k=1}^{m} \left(1-r_{jk}\right), j=1,2,\cdots,m$$

Where:$r_{jk}$ is the correlation coefficient used to evaluate the correlation between indicator *j* and indicator *k*, as follows:

$$r_{jk}=\frac{Cov\left(x,y\right)}{\sigma_{j}\sigma_{k}}$$

3) Calculate the combined information content of the indicators. That is, multiply the indicator variability by the correlation.

$$C_{j}=\sigma_{j}R_{j}, j=1,2,\cdots,m$$

4) Determination of indicator weights. The amount of information obtained for the indicators is normalized, and the final weights are obtained.

$$w_{j}=\frac{C_{j}}{\sum_{k=1}^{m} C_{k}},j=1,2,\cdots,m$$

However, the single CRITIC method fails to take into account the dispersion between indicators, and the entropy weight method can effectively compensate for this deficiency.

**3. Entropy weight method**

Entropy weighting is also an objective weighting method, the concept of entropy is derived from thermodynamics, and its principle is to determine the weights according to the size of the information provided by each indicator, which avoids the subjectivity of human judgement^[2]^. The smaller the entropy value, the greater the amount of information contained, that is, the greater the degree of variability of the indicator value, the greater the role of the indicator in the comprehensive evaluation, the greater the corresponding weight. The specific steps of applying the entropy weight method to determine the weights are as follows:

1) Calculate the information entropy of each evaluation indicator. The information entropy of the *jth* indicator is shown in the following equation.

$$E_{j}=-k\sum_{i=1}^{m} f_{ij}\ln f_{ij}, i,j=1,2,\cdots m$$

$$f_{ij}=b_{ij}/\sum_{i=1}^{m} b_{ij}$$

Where$k=\frac{1}{\ln m}$ , and can be known as$0\leq E_{j}\leq1$ . Where$f_{ij}$ is the weight of the *jth* indicator in the *ith* evaluation object.

2) Calculate the entropy weight of each evaluation indicator, and the weight of the *jth* indicator is shown in the following equation.

$$w'_{j}=\frac{1-E_{j}}{n-\sum_{j=1}^{n} E_{j}}, j=1,2,\cdots,n$$

**4. Analytic hierarchy process**

Analytic Hierarchy Process (AHP), is a theoretical method designed to solve multi-objective decision-making problems by quantifying information through expert scoring. The main steps in determining the weights using AHP are as follows.

1) Scale determination and construction of judgement matrices

In this paper, the comparative judgement matrix is constructed with the help of 9-scale method^[4]^, and the meanings of the scales are shown in **Table A1**. By comparing the pairwise relationship between the influencing factors of the same level, the comparison matrix of each level is constructed from the top down to form the judgement matrix$A=\left(a_{ij}\right)_{m\times n}$ , where$a_{ij}$ represents the ratio of the importance of factor *i* and factor *j*.

**Table A1**

Meaning of the 1-9 scale

| scale | Importance comparison of factors *i*, *j* |
| --- | --- |
| 1 | Indicates that factor *i* has the same importance as factor *j* |
| 3 | Indicates that factor *i* is slightly more important than factor *j* |
| 5 | indicates that factor *i* is significantly more important than factor *j* |
| 7 | Indicates that factor *i* is more strongly important than factor *j* |
| 9 | Indicates extreme importance of factor *i* over factor *j* |
| 2, 4, 6, 8 | Indicates that the importance of the two factors is between the importance of the neighboring scales above |
| The inverse of the above value | If the ratio of the importance of factor *i* to factor *j* is$a_{ij}$ , then the ratio of the importance of factor *j* to factor *i* is$1/a_{ij}$ |

2) Weight calculation and consistency test

Sum each column of matrix A, and then normalize each element by column to obtain the standard matrix; sum the standard matrix by rows to obtain the vector *w*; normalize the vector *w* to obtain the weight matrix *W*; according to the formula$AW=\lambda_{\max}W$ , the maximum eigenvalue$\lambda_{\max}$ of the judgement matrix A can be obtained.

Calculate the consistency indicator CI, see the following equation:

$$CI=\frac{\lambda_{\max}-n}{n-1}$$

The larger the CI value, the more the matrix deviates from perfect consistency; the closer the CI value tends to zero, the closer the matrix is to perfect consistency.

Calculate the consistency ratio CR, see the following equation:

$$CR=\frac{CI}{RI}$$

It is stipulated that if CR < 0.1, the consistency of the matrix is acceptable; conversely, it indicates that the weighting coefficients are not properly assigned and need to be adjusted appropriately; where *RI* is the random consistency index, as shown in **Table A2**.

**Table A2**

RI values

| Matrix order n | 1 | 2 | 3 | 4 | 5 | 6 | 7 | 8 | 9 |
| --- | --- | --- | --- | --- | --- | --- | --- | --- | --- |
| *RI* | 0 | 0 | 0.58 | 0.9 | 1.12 | 1.24 | 1.32 | 1.41 | 1.45 |

**5. TOPSIS-based ranking methods**

The TOPSIS method is a multi-indicator decision analysis method that can be used to rank the strengths and weaknesses of a limited number of evaluation objects. Based on the literature^[6]^, the calculation steps are as follows:

***Step 1*** Use the weight information obtained by the game theory combination weighting method to weight the standardized matrix, construct the weighted decision matrix, and select the positive-ideal solution $Z^{+}$ and the negative-ideal solution $Z^{-}$. The positive-ideal solution $Z^{+}$ is a vector of the maximum values of all indicators, and the negative-ideal solution $Z^{-}$ is a vector of the minimum values of all indicators.

***Step 2*** Calculate the Euclidean distance between each assessed object and both the positive and negative-ideal solutions.

$$D_{i}^{+}=\sqrt{\sum_{i=1}^{n} \left(Z_{ij}-Z^{+}\right)^{2}}$$

$$D_{i}^{-}=\sqrt{\sum_{i=1}^{n} \left(Z_{ij}-Z^{-}\right)^{2}}$$

***Step 3*** Calculate the proximity of each evaluation object $C_{i}$ . Finally, the evaluation objects are ranked to determine the optimal solution. The value range of $C_{i}$ is 0~1, the closer to 1, the better the evaluation object.

$$C_{i}=\frac{D_{i}^{-}}{D_{i}^{+}+D_{i}^{-}}$$

## Reference

[1] Li, W., Dong, F., et al. (2023). Evaluation of provincial power supply reliability with high penetration of renewable energy based on combination weighting of game theory-TOPSIS method. Sustainable Energy, Grids and Networks, 35, 101092.
[2] Li, S., Wei, H., et al. (2014). Evaluation of urban human settlement quality in Ningxia based on analytic hierarchy process and entropy weight method. Chinese Journal of Applied Ecology, 25(9), 2700-2708.
[3] Diakoulaki, D., Mavrotas, G., Papayannakis, L. (1995). Determining objective weights in multiple criteria problems: The critic method. Computers & Operations Research, 22(7), 763-770.
[4] Ding, X., Chong, X., et al. (2017). Fuzzy comprehensive assessment method based on the entropy weight method and its application in the water environmental safety evaluation of the Heshangshan drinking water source area, three gorges reservoir area, China. Water, 9(5), 329.
[5] Wu, Y. H., Zhou, X. L., Li, G. H. (2022). Construction and application of a dynamic bidirectional evaluation index system for regional economic resilience in China under the new development pattern. Academic Exchange, (8), 98-111+192.
[6] Sun, C. C. (2010). A performance evaluation model by integrating fuzzy AHP and fuzzy TOPSIS methods. Expert systems with applications, 37(12), 7745-7754.
