# Supplementary material for: Indicator system for regional public health emergency response capacity based on ternary space theory: construction and empirical study in China
Source: BMC Public Health. 2026 Aug 1;26:3330. doi: 10.1186/s12889-026-28703-4 (PMC13753278; doi:10.1186/s12889-026-28703-4)
Supplement: Supplementary file 2 [file 12889_2026_28703_MOESM2_ESM.docx]

**Appendix B. Full list of indicator weights**

**Table B1** Weights of the evaluation indicator system

| Level 1 indicators | Level 2 indicators | Level 3 indicators | AHP weight (%) | CRITIC weight (%) | Entropy weight (%) | Combined weight (%) |
| --- | --- | --- | --- | --- | --- | --- |
| Pre-preparation (29.73) | Physical space (33.33) | Centers for Disease Control and Prevention(CDC) per 10,000 population | 2.48 | 2.4 | 4.02 | 3.11 |
|  |  | Agglomeration degree of CDC | 3.77 | 2.39 | 2.28 | 2.97 |
|  |  | Share of health supervision institute in professional public health institutions | 1.03 | 3.34 | 1.05 | 1.33 |
|  |  | Share of level A tertiary hospitals in the number of hospitals | 1.52 | 2.72 | 1.2 | 1.54 |
|  |  | Number of beds in preventive health departments per 10,000 population | 0.45 | 2.12 | 4.74 | 2.46 |
|  |  | Agglomeration degree of beds in preventive health departments | 0.66 | 1.24 | 6.94 | 3.36 |
|  | Social space  (33.33) | CDC personnel per 10,000 population | 2.35 | 2.39 | 1.64 | 2.06 |
|  |  | Agglomeration degree of CDC personnel | 3.47 | 2.13 | 4.61 | 3.78 |
|  |  | Annual health check-ups as a proportion of the population | 1.05 | 1.18 | 1.57 | 1.28 |
|  |  | Number of promotional materials per 10,000 population | 0.32 | 1.5 | 1.48 | 0.96 |
|  |  | Number of public health education activities per 10,000 population | 0.69 | 1.91 | 3.42 | 1.99 |
|  |  | Share of medical expenditure in fiscal expenditure | 1.58 | 2.89 | 0.57 | 1.32 |
|  |  | Number of family health services per 10,000 population | 0.46 | 1.47 | 3.26 | 1.76 |
|  | Information space  (33.33) | Coverage of websites hosted by health education professional bodies | 2.48 | 1.55 | 1.55 | 1.97 |
|  |  | Health Education Column Coverage | 7.43 | 2.23 | 1.7 | 4.36 |
| In-process response (53.90) | Physical space (33.33) | Number of emergency medicine beds per 10,000 population | 5.87 | 2.09 | 1.84 | 3.70 |
|  |  | Number of infectious disease beds per 10,000 population | 4.08 | 2.95 | 1.73 | 2.95 |
|  |  | Hospitals per 10,000 population | 1.32 | 2.77 | 1.2 | 1.46 |
|  |  | Agglomeration degree of hospital | 1.93 | 2.23 | 4.04 | 2.85 |
|  |  | Number of beds in other hospital departments per 10,000 population | 0.43 | 2.47 | 0.95 | 0.91 |
|  |  | Number of beds in primary healthcare institutions per 10,000 population | 0.89 | 3.4 | 0.82 | 1.19 |
|  |  | Number of primary healthcare institutions as a proportion of the number of grassroots organizations in urban and rural areas | 0.61 | 1 | 1.29 | 0.95 |
|  |  | Transport network density | 2.82 | 3.23 | 1.46 | 2.30 |
|  | Social space  (33.33) | The utilization rate of hospital beds | 0.43 | 2.24 | 1.05 | 0.92 |
|  |  | The daily average workload of patient visits of physicians in the hospital | 0.61 | 3.52 | 0.51 | 0.94 |
|  |  | Primary healthcare institutions personnel per 10,000 population | 1.32 | 1.96 | 0.82 | 1.19 |
|  |  | Agglomeration degree of personnel in primary healthcare institutions | 1.93 | 1.83 | 4.54 | 3.01 |
|  |  | Hospital practicing physician (assistants) per 10,000 population | 5.87 | 1.75 | 1.24 | 3.40 |
|  |  | Hospital registered nurses per 10,000 population | 4.08 | 1.65 | 0.51 | 2.27 |
|  |  | Agglomeration degree of personnel in hospital | 2.82 | 2.08 | 5.88 | 4.01 |
|  |  | Strength of leadership of grassroots organizations | 0.89 | 2.07 | 1.98 | 1.50 |
|  | Information space  (33.33) | Internet penetration rate | 13.47 | 2.33 | 0.98 | 6.81 |
|  |  | Telephone penetration rate | 4.49 | 2.03 | 1.08 | 2.75 |
| Post-recovery (16.38) | Physical space (33.33) | Rehabilitation medicine beds per 10,000 population | 1.51 | 2.43 | 1.06 | 1.44 |
|  |  | Agglomeration degree of beds in Rehabilitation medicine departments | 2.54 | 1.76 | 6.15 | 3.95 |
|  |  | Psychiatric beds per 10,000 population | 0.52 | 2.34 | 1.27 | 1.07 |
|  |  | Agglomeration degree of beds in Psychiatric departments | 0.88 | 1.44 | 7.28 | 3.63 |
|  | Social space  (33.33) | Share of urban residents' healthcare expenditure in consumer expenditure | 0.56 | 2.6 | 0.63 | 0.85 |
|  |  | Share of rural residents' healthcare expenditure in consumer expenditure | 0.56 | 2.2 | 0.38 | 0.70 |
|  |  | Number of technical consultations and policy recommendations | 0.25 | 1.44 | 4.58 | 2.22 |
|  |  | Number of medical aid funds per capita for subsidized persons | 0.94 | 2.17 | 3.06 | 1.99 |
|  |  | The incidence rate of class A and B notifiable infectious diseases (1/100,000) | 1.58 | 3.63 | 0.81 | 1.52 |
|  |  | The mortality rate of class A and B notifiable infectious diseases (1/100,000) | 1.58 | 3.92 | 0.65 | 1.49 |
|  | Information space  (33.33) | Share of science and technology expenditures in fiscal expenditures | 5.46 | 2.98 | 2.18 | 3.77 |
