# Supplementary material for: Indicator system for regional public health emergency response capacity based on ternary space theory: construction and empirical study in China
Source: BMC Public Health. 2026 Aug 1;26:3330. doi: 10.1186/s12889-026-28703-4 (PMC13753278; doi:10.1186/s12889-026-28703-4)
Supplement: Supplementary file 3 [file 12889_2026_28703_MOESM3_ESM.docx]

**Appendix C. Longitudinal Analysis (2013-2021)**

**Table C1** Relative proximity of public health emergency response capacity by province in the eastern region, 2013-2021

| Year | Shanghai | Bei  jing | Tian  jin | Shan  dong | Guangdong | Jiang  su | Hebei | Zhe  jiang | Hai  nan | Fujian | Liao  ning |
| --- | --- | --- | --- | --- | --- | --- | --- | --- | --- | --- | --- |
| 2013 | 0.505 | 0.343 | 0.470 | 0.355 | 0.284 | 0.331 | 0.129 | 0.290 | 0.211 | 0.464 | 0.463 |
| 2014 | 0.462 | 0.410 | 0.480 | 0.415 | 0.251 | 0.334 | 0.244 | 0.302 | 0.227 | 0.539 | 0.506 |
| 2015 | 0.434 | 0.359 | 0.508 | 0.408 | 0.380 | 0.347 | 0.183 | 0.351 | 0.255 | 0.532 | 0.556 |
| 2016 | 0.463 | 0.409 | 0.591 | 0.440 | 0.505 | 0.386 | 0.279 | 0.431 | 0.341 | 0.548 | 0.574 |
| 2017 | 0.469 | 0.546 | 0.552 | 0.504 | 0.537 | 0.417 | 0.404 | 0.382 | 0.295 | 0.590 | 0.635 |
| 2018 | 0.472 | 0.532 | 0.597 | 0.573 | 0.643 | 0.457 | 0.519 | 0.408 | 0.316 | 0.615 | 0.664 |
| 2019 | 0.471 | 0.586 | 0.601 | 0.654 | 0.672 | 0.492 | 0.590 | 0.444 | 0.365 | 0.639 | 0.646 |
| 2020 | 0.508 | 0.642 | 0.442 | 0.550 | 0.560 | 0.406 | 0.677 | 0.362 | 0.366 | 0.453 | 0.474 |
| 2021 | 0.532 | 0.629 | 0.680 | 0.763 | 0.713 | 0.863 | 0.787 | 0.758 | 0.716 | 0.746 | 0.740 |

**Table C2** Relative proximity of public health emergency response capacity by province in the central region, 2013-2021

| Year | Jilin | Anhui | Shanxi | Jiangxi | Henan | Hubei | Hunan | Heilongjiang |
| --- | --- | --- | --- | --- | --- | --- | --- | --- |
| 2013 | 0.294 | 0.202 | 0.38 | 0.175 | 0.141 | 0.233 | 0.23 | 0.309 |
| 2014 | 0.423 | 0.22 | 0.384 | 0.21 | 0.183 | 0.281 | 0.268 | 0.339 |
| 2015 | 0.338 | 0.228 | 0.402 | 0.244 | 0.18 | 0.303 | 0.282 | 0.368 |
| 2016 | 0.394 | 0.373 | 0.414 | 0.301 | 0.223 | 0.374 | 0.368 | 0.404 |
| 2017 | 0.445 | 0.443 | 0.421 | 0.408 | 0.277 | 0.497 | 0.454 | 0.47 |
| 2018 | 0.515 | 0.543 | 0.452 | 0.526 | 0.34 | 0.613 | 0.574 | 0.543 |
| 2019 | 0.557 | 0.676 | 0.528 | 0.669 | 0.418 | 0.712 | 0.697 | 0.653 |
| 2020 | 0.538 | 0.676 | 0.511 | 0.738 | 0.47 | 0.713 | 0.72 | 0.601 |
| 2021 | 0.674 | 0.953 | 0.802 | 0.929 | 0.905 | 0.811 | 0.847 | 0.826 |

**Table C3** Relative proximity of public health emergency response capacity by province in the western region, 2013-2021

| Year | Yunnan | Inner Mongolia | Sichuan | Ningxia | Guangxi | Xinjiang | Gansu | Tibet | Guizhou | Chongqing | Shaanxi | Qinghai |
| --- | --- | --- | --- | --- | --- | --- | --- | --- | --- | --- | --- | --- |
| 2013 | 0.234 | 0.377 | 0.106 | 0.325 | 0.202 | 0.166 | 0.331 | 0.386 | 0.251 | 0.279 | 0.129 | 0.188 |
| 2014 | 0.281 | 0.406 | 0.15 | 0.328 | 0.225 | 0.17 | 0.347 | 0.372 | 0.291 | 0.321 | 0.302 | 0.224 |
| 2015 | 0.3 | 0.447 | 0.219 | 0.393 | 0.278 | 0.278 | 0.392 | 0.408 | 0.323 | 0.405 | 0.376 | 0.377 |
| 2016 | 0.341 | 0.488 | 0.305 | 0.459 | 0.333 | 0.317 | 0.426 | 0.431 | 0.383 | 0.489 | 0.343 | 0.39 |
| 2017 | 0.395 | 0.533 | 0.389 | 0.511 | 0.448 | 0.265 | 0.509 | 0.461 | 0.479 | 0.591 | 0.427 | 0.389 |
| 2018 | 0.459 | 0.574 | 0.471 | 0.525 | 0.566 | 0.42 | 0.615 | 0.476 | 0.566 | 0.698 | 0.495 | 0.427 |
| 2019 | 0.492 | 0.64 | 0.601 | 0.527 | 0.648 | 0.395 | 0.696 | 0.523 | 0.669 | 0.753 | 0.586 | 0.594 |
| 2020 | 0.494 | 0.55 | 0.614 | 0.457 | 0.735 | 0.438 | 0.592 | 0.484 | 0.653 | 0.668 | 0.597 | 0.609 |
| 2021 | 0.815 | 0.726 | 0.78 | 0.78 | 0.813 | 0.845 | 0.85 | 0.692 | 0.848 | 0.848 | 0.707 | 0.818 |
